# A 5:2 Intermittent Fasting Meal Replacement Diet and Glycemic Control for Adults With Diabetes: The EARLY Randomized Clinical Trial

**DOI:** 10.1001/jamanetworkopen.2024.16786

**Published:** 2024-06-21

**Authors:** Lixin Guo, Yue Xi, Wenbo Jin, Huijuan Yuan, Guijun Qin, Shuchun Chen, Lihui Zhang, Yu Liu, Xingbo Cheng, Wen Liu, Dongni Yu

**Affiliations:** 1 Department of Endocrinology, Beijing Hospital, National Center of Gerontology, Institute of Geriatric Medicine, Chinese Academy of Medical Sciences, Beijing, China; 2 Department of Endocrinology, The Third Affiliated Hospital of Jinzhou Medical University, Jinzhou, Liaoning, China; 3 Department of Endocrinology, Nanyang Central Hospital, Nanyang, Henan, China; 4 Department of Endocrinology, Henan Provincial People’s Hospital, Zhengzhou, Henan, China; 5 Department of Endocrinology, The First Affiliated Hospital of Zhengzhou University, Zhengzhou, Henan, China; 6 Department of Endocrinology, Hebei Provincial People’s Hospital, Shijiazhuang, Hebei, China; 7 Department of Endocrinology, The Second Hospital of Hebei Medical University, Shijiazhuang, Hebei, China; 8 Department of Endocrinology, Sir Run Run Hospital, Nanjing Medical University, Nanjing, China; 9 Department of Endocrinology, The First Affiliated Hospital of Soochow University, Soochow, Jiangsu, China; 10 Department of Clinical Nutrition, Beijing Tongren Hospital, Capital Medical University, Beijing, China

## Abstract

**Importance:**

An intermittent fasting plan consisting of 2 nonconsecutive fasting days and 5 days of habitual intake per week and meal replacement diet (5:2 MR) could provide additional benefits to patients with type 2 diabetes.

**Objective:**

To evaluate the effect of the 5:2 MR on glycemic control among patients with early type 2 diabetes compared with metformin and empagliflozin.

**Design, Setting, and Participants:**

The EARLY (Exploration of Treatment of Newly Diagnosed Overweight/Obese Type 2 Diabetes Mellitus) study is a randomized, open-label, active parallel-controlled clinical trial conducted between November 13, 2020, and December 29, 2022, in 9 centers across China. A total of 509 eligible patients underwent screening, out of which 405 were randomly assigned to 3 groups and included in the intention-to-treat analysis.

**Interventions:**

Patients were randomly allocated in a 1:1:1 ratio to receive either metformin, empagliflozin, or 5:2 MR. The treatment was 16 weeks, with an 8-week follow-up.

**Main Outcomes and Measures:**

The primary end point was the change in hemoglobin A_1c_ (HbA_1c_) level from baseline to 16 weeks. Secondary end points included changes in body weight, anthropometric measurements, and biochemical parameters.

**Results:**

Of the 405 randomized participants (265 men [65.4%]; mean [SD] age, 45.5 [11.0] years; mean [SD] body mass index, 29.5 [4.1]; and mean [SD] HbA_1c_ level, 7.9% [0.6%]), 332 completed the 16-week treatment. From baseline to week 16, participants in the 5:2 MR group showed the greatest reduction in HbA_1c_ (least-squares mean [LSM], −1.9% [SE, 0.2%]), significantly greater than patients receiving metformin (LSM, −1.6% [SE, 0.2%]; adjusted LSM difference, −0.3% [95% CI, −0.4% to −0.1%]) and empagliflozin (LSM, −1.5% [SE, 0.2%]; adjusted LSM difference, −0.4% [95% CI, −0.6% to −0.2%]). At week 16, the mean weight loss in the 5:2 MR group (LSM, −9.7 kg [SE, 2.2 kg]) was greater than that in the metformin group (LSM, −5.5 kg [SE, 2.3 kg]) and empagliflozin group (LSM, −5.8 kg [SE, 2.3 kg]).

**Conclusions and Relevance:**

This randomized clinical trial of Chinese adults with overweight or obesity and with early type 2 diabetes found that 5:2 MR could improve glycemic outcomes and weight loss in the short term compared with metformin or empagliflozin, making it a promising initial intervention and early management for type 2 diabetes.

**Trial Registration:**

Chinese Clinical Trial Registry Identifier: ChiCTR2000040656

## Key Points

**Question:**

What is the effect of a 16-week intermittent fasting plan consisting of 2 nonconsecutive fasting days and 5 days of habitual intake per week and meal replacement diet (5:2 MR) on the changes in hemoglobin A_1c_ level in Chinese adults with early type 2 diabetes?

**Findings:**

In this randomized clinical trial of 405 adults, the 5:2 MR approach achieved better glycemic control at 16 weeks compared with metformin and empagliflozin.

**Meaning:**

The 5:2 MR approach may serve as an effective initial lifestyle intervention instead of antidiabetic drugs for patients with type 2 diabetes.

## Teaser

This randomized clinical trial evaluates the effect of 16 weeks of intermittent fasting plus a meal replacement diet on glycemic control among patients with early type 2 diabetes compared with metformin and empagliflozin.

## Introduction

The latest data from the International Diabetes Federation in 2021 reveal that there are 537 million adults with diabetes globally, affecting approximately 1 in 10 adults.^1^ China has the highest number of adults with diabetes in the world; from 2011 to 2021, the number increased from 90 million to 140.9 million, a 56.6% increase.^1^ The prevalence of diabetes among Chinese adults is 12.4%.^2^ According to China standards,^3^ about half the population is either overweight (body mass index [BMI; calculated as weight in kilograms divided by height in meters squared] 24-27.9) or obese (BMI ≥28).^4^

Overweight and obesity are significant risk factors for the development of type 2 diabetes.^5,6,7^ Appropriate weight loss can improve glycemic control and reduce the dosage of antidiabetic drugs among patients with type 2 diabetes.^8^ However, achieving weight loss is often challenging, necessitating the implementation of strategies such as meal replacement (MR) or dietary restriction.

Meal replacement is a prepackaged food or beverage that is substituted for 1 or more meals and provides energy.^9^ The Look AHEAD study has demonstrated that, as part of a comprehensive lifestyle intervention, at 1 year MR effectively reduced hemoglobin A_1c_ (HbA_1c_) levels by 0.7% (to convert to proportion of total hemoglobin, multiply by 0.01) and achieved initial weight loss of 8.6% to 9.0% among patients with overweight or obesity and type 2 diabetes.^10,11^ A systematic review including 23 studies and 7884 adults found that MR was associated with more weight loss (mean, −1.4 kg [95% CI −2.5 to −0.4 kg]) compared with other diets.^12^ Important randomized clinical trials in White European indviduals (DiRECT),^13^ Middle Eastern indviduals (DIADEM-I)^14^ and South Asian individuals (STANDby)^15^ have proved that MR can alleviate diabetes by lowering body weight.

As a dietary therapy, the 5:2 intermittent fasting diet involves 2 nonconsecutive fasting days (one-fourth the energy intake of habitual diet) and 5 days of habitual intake per week.^16^ Individuals with obesity have successfully lost weight with this diet through both short-term and long-term interventions.^17,18,19^ A single-center randomized clinical trial with a small sample size of 137 participants found that a 12-month 5:2 intermittent fasting diet significantly decreased HbA_1c_ levels among patients with overweight or obesity and type 2 diabetes, compared with a continuous energy restriction diet.^20^

Combining the 5:2 intermittent fasting diet with MR (5:2 MR) could provide additional benefits to patients and is worthy of investigation. We aimed to investigate the efficacy of 16 weeks of 5:2 MR on HbA_1c_ changes among Chinese adults with overweight or obesity and early-stage type 2 diabetes.

## Methods

### Study Design and Participants

The EARLY (Exploration of Treatment of Newly Diagnosed Overweight/Obese Type 2 Diabetes Mellitus) study is a randomized, open-label, active parallel-controlled clinical trial. The study protocol (Supplement 1) was approved by the ethics committees of all participating centers (Beijing Hospital; the Third Affiliated Hospital of Jinzhou Medical University; Nanyang Central Hospital; Henan Provincial People’s Hospital; the First Affiliated Hospital of Zhengzhou University; Hebei Provincial People’s Hospital; the Second Hospital of Hebei Medical University; Sir Run Run Hospital, Nanjing Medical University; and the First Affiliated Hospital of Soochow University). The trial followed the International Conference on Harmonization Guidelines for Good Clinical Practice and the Declaration of Helsinki.^21^ All patients provided written informed consent. This report adhered to the Consolidated Standards of Reporting Trials (CONSORT) reporting guideline.

### Eligibility Criteria and Recruitment

We recruited adults with newly diagnosed (within 1 year) type 2 diabetes who had not used antidiabetic agents in the past 3 months, aged 18 to 65 years, with a BMI of 24 or more and an HbA_1c_ level of 7% to 9%. The recruitment was conducted concurrently at 9 hospitals across China (eTable 1 in Supplement 2) from November 13, 2020, to December 29, 2022. We excluded participants who had used weight-loss drugs or products within the past 3 months before enrollment, as well as pregnant or breastfeeding women (Supplement 1).

### Randomization and Masking

Randomization was conducted using an interactive web response system. The randomization list of participants was generated by the stratified blocked randomization method using SAS software, version 9.4 (SAS Institute Inc), in which stratification was based on the center (block size of 9). Within each stratum, participants were randomized using a block randomization method, with a block size of 9, in a ratio of 1:1:1 to receive either metformin, empagliflozin, or 5:2 MR. Both the lists for participant and treatment allocation were inputted into the interactive web response system. At the study site, participants were administered treatment based on the randomization code and the corresponding treatment group obtained from the interactive web response system. Due to the nature of the intervention, blinding of participants and investigators was not feasible in this study. However, during the data analysis, the statisticians remained blinded to the study groupings.

### Interventions

The treatment period lasted for 16 weeks, followed by an 8-week follow-up (eFigure 1 in Supplement 2). All participants received dietary and exercise guidance as well as general diabetes education from nutritionists and research physicians in accordance with China Guideline^22^ (eTable 2 in Supplement 2) every 4 weeks.

### 5:2 MR Approach

Patients in the 5:2 MR group consumed low-energy MR product A (Kang zhijun, Beijing MetabolicControl Technology Co Ltd; eTable 3 in Supplement 2). The 5:2 MR approach (eFigure 2 in Supplement 2) means that, within 1 week, there were 2 nonconsecutive days on which meals are replaced. On these 2 days, participants were required to consume 1 serving of Kang zhijun A instead of all 3 regular meals, with a daily energy intake of 500 kcal for women and 600 kcal for men. On the remaining 5 days, participants chose their own breakfast and lunch but had 1 serving of Kang zhijun B for dinner and were encouraged to monitor their calorie intake. Throughout the 16 weeks, dietary intake was recorded in a diary.

### Administration of Metformin or Empagliflozin

Patients took metformin (Shanghai Bristol-Myers Squibb), 0.5 g, twice a day. If the initial drug dosage was well tolerated, it was escalated to 2 g per day. Empagliflozin (Shanghai Boehringer Ingelheim), 10 mg, was administered once a day. During the study, patients were instructed to promptly contact the research center’s physician in case of severe hypoglycemia.

### Outcomes

The primary outcome was the change in HbA_1c_ level from baseline to 16 weeks. Secondary outcomes included changes in weight (measured by InBody 770 [InBody]), BMI, waist circumference, hip circumference, waist to hip ratio, systolic and diastolic blood pressure, fasting plasma glucose (FPG) level, fasting insulin level, fasting C-peptide level, homeostasis model assessment of insulin resistance (HOMA-IR = FPG [mmol/L] × fasting insulin [μU/mL]/22.5), lipid profiles (total cholesterol, triglycerides, high-density lipoprotein cholesterol [HDL-C], and low-density lipoprotein cholesterollevels), and uric acid levels. The primary and secondary outcomes were reevaluated at the end of 8-week follow-up (week 24).

Adverse events were assessed throughout the study. Adverse events of particular interest included gastrointestinal reactions, urinary tract and reproductive system infections, hypoglycemia, and hyperglycemia. Laboratory testing was conducted at a central laboratory.

### Statistical Analysis

The sample size calculation was based on the SD of the change in HbA_1c_ level from a previous study,^23^ with a 2-sided α of .05, β of 0.2, a minimum detectable between-group difference of 0.1%, and an anticipated SD of 0.2% based on pilot data analysis and a multiple pairwise comparison test using the Tukey-Kramer test. It was computed that each group required 108 participants using PASS 15 software (NCSS). Accounting for an expected 20% dropout rate, each group required 135 patients.

The primary outcome was analyzed following the intention-to-treat principle in the full analysis set, which included all randomized participants who received at least 1 dose of drugs or 5:2 MR. The safety outcome was analyzed in the safety analysis set, defined as participants randomized who received at least 1 dose of drugs or 5:2 MR and had safety assessment data collected at least once after the baseline.

The primary outcome was analyzed using the analysis of covariance model, which calculated the least-squares mean (LSM) and 95% CI to compare changes in HbA_1c_ level and key secondary outcomes among the 3 groups. The model adjusted for sex, age, height, weight, family history of diabetes and hypertension, physical activity, smoking, alcohol consumption, and baseline HbA_1c_. Multiple imputation was used for missing values in the primary and key secondary outcomes (eMethods in Supplement 2). Post hoc subgroup analyses were conducted to explore the potential effect of baseline differences on HbA_1c_ and weight loss. Statistical analyses were performed using SPSS, version 24.0 software (SPSS Inc). All *P* values were from 2-sided tests and results were deemed statistically significant at *P* < .05.

## Results

### Baseline Characteristics

**Figure 1.**
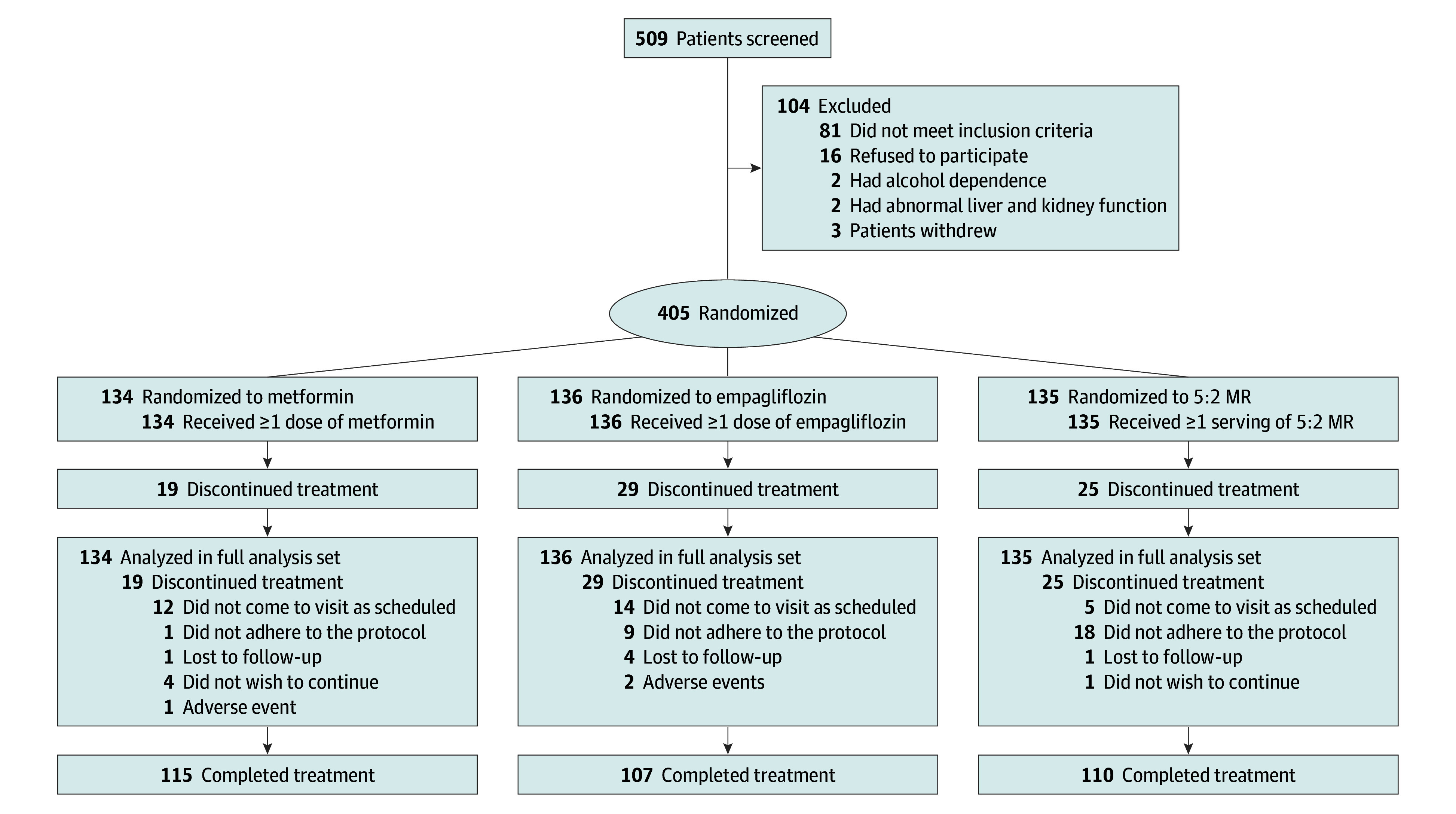
CONSORT Flow Diagram 5:2 MR indicates an intermittent fasting plan consisting of 2 nonconsecutive fasting days and 5 days of habitual intake per week and meal replacement diet.

Of the 509 participants screened, 405 adults with type 2 diabetes (265 men [65.4%] and 140 women [34.6%]; mean [SD] age, 45.5 [11.0] years; mean [SD] BMI, 29.5 [4.1]; mean [SD] HbA_1c_ level, 7.9% [0.6%]) were randomly allocated. The patients’ baseline characteristics are presented in Table 1. Of these 405 participants, 134 were randomized to the metformin group, 136 to the empagliflozin group, and 135 to the 5:2 MR group, all included in the intention-to-treat analysis (Figure 1). Finally, 332 patients completed the 16-week treatment, for a completion rate of 82.0%.

**Table 1.** Baseline Characteristics of the Study Participants

Characteristic	Metformin (n = 134)	Empagliflozin (n = 136)	5:2 MR (n = 135)	Total (N = 405)	Standardized differences		
					5:2 MR vs metformin	5:2 MR vs empagliflozin	Metformin vs empagliflozin
Age, mean (SD), y	46.8 (11.4)	46.5 (10.3)	43.3 (10.8)	45.5 (11.0)	0.31	0.30	0.03
Sex, No. (%)							
Male	89 (66.4)	95 (69.9)	81 (60.0)	265 (65.4)	0.13	0.21	0.08
Female	45 (33.6)	41 (30.1)	54 (40.0)	140 (34.6)	0.13	0.21	0.08
Current smoker, No. (%)	45 (33.8)	60 (44.1)	48 (35.6)	153 (37.9)	0.04	0.21	0.21
Current drinker, No. (%)	49 (36.8)	45 (33.1)	50 (37.0)	144 (35.6)	0	0.08	0.08
Menopausal status, No. (%)^a^	16 (53.3)	15 (44.1)	23 (54.8)	54 (50.9)	0.03	0.18	0.18
Family history of diabetes, No. (%)	66 (49.6)	70 (51.5)	61 (45.2)	197 (48.8)	0.09	0.04	0.04
Family history of hypertension, No. (%)	46 (34.6)	58 (42.6)	60 (44.4)	164 (40.6)	0.20	0.16	0.16
Comorbidities at screening, No. (%)							
Hypertension	38 (28.6)	56 (41.2)	47 (34.8)	141 (34.9)	0.13	0.27	0.27
Dyslipidemia	37 (27.8)	41 (30.1)	34 (25.2)	112 (27.7)	0.06	0.05	0.05
Coronary heart disease	4 (3.0)	6 (4.4)	3 (2.2)	13 (3.2)	0.05	0.07	0.07
Physical activity, No. (%)							
Inactivity	22 (15.8)	26 (19.1)	13 (9.6)	60 (14.9)	0.19	0.09	0.09
<150 min/wk	90 (67.7)	86 (63.2)	93 (68.9)	269 (66.6)	0.03	0.09	0.09
≥150 min/wk	22 (16.5)	24 (17.6)	29 (21.5)	75 (18.6)	0.11	0.03	0.03
Carbohydrate-based foods, mean (SD), g	298.9 (115.6)	301.5 (115.5)	323.9 (138.6)	308.1 (123.9)	0.20	0.18	0.02
Body weight, mean (SD), kg	82.0 (15.0)	84.9 (14.3)	87.0 (16.9)	84.6 (15.6)	0.32	0.14	0.20
BMI, mean (SD)	28.9 (3.8)	29.5 (3.9)	30.2 (4.4)	29.5 (4.1)	0.31	0.16	0.15
BMI range, No. (%)							
24 to <28	74 (55.2)	60 (44.1)	53 (39.3)	187 (46.2)	0.32	0.22	0.22
≥28	60 (44.8)	76 (55.9)	82 (60.7)	218 (53.8)	0.32	0.22	0.22
Waist circumference, mean (SD), cm	98.6 (9.7)	99.3 (10.1)	100.9 (11.2)	99.6 (10.4)	0.22	0.15	0.08
Hip circumference, mean (SD), cm	104.6 (8.1)	105.1 (8.0)	106.6 (8.4)	105.4 (8.2)	0.25	0.19	0.06
Waist to hip ratio, mean (SD)	0.9 (0.1)	0.9 (0.1)	1.0 (0.1)	0.9 (0.1)	0.12	0.12	0
HbA_1c_, mean (SD), %	7.9 (0.6)	7.9 (0.6)	7.8 (0.5)	7.9 (0.6)	0.09	0.04	0.05
Fasting plasma glucose, mean (SD), mg/dL	149.9 (37.1)	147.6 (31.7)	146.3 (35.0)	147.9 (34.6)	0.10	0.04	0.07
Fasting insulin, mean (SD), μIU/mL	33.9 (41.2)	33.4 (34.7)	41.4 (55.3)	36.2 (44.6)	0.15	0.17	0.01
Fasting C-peptide, mean (SD), ng/mL	2.6 (1.7)	2.8 (3.4)	2.6 (1.5)	2.7 (2.3)	0.02	0.05	0.06
HOMA-IR, mean (SD)	13.7 (18.4)	13.1 (14.7)	16.4 (24.7)	14.4 (19.7)	0.12	0.16	0.04
Blood pressure, mean (SD), mm Hg							
Systolic	131 (14)	131 (15)	131 (15)	131 (15)	0.01	0	0.01
Diastolic	82 (10)	82 (10)	83 (11)	82 (10)	0.11	0.07	0.04
Lipids, mean (SD), mg/dL							
Total cholesterol	201.2 (44.0)	198.1 (64.5)	193.1 (41.3)	197.3 (51.0)	0.19	0.09	0.06
Triglycerides	230.1 (236.3)	218.6 (308.9)	191.2 (164.6)	213.3 (243.4)	0.19	0.11	0.04
LDL-C	121.6 (34.4)	118.9 (40.9)	118.5 (33.6)	119.7 (36.3)	0.09	0.01	0.07
HDL-C	44.0 (10.4)	42.1 (8.1)	42.1 (8.5)	42.9 (9.3)	0.20	0	0.21
Uric acid	5689.0 (1857.3)	5920.7 (1622.2)	5852.7 (1929.4)	5821.8 (1805.5)	0.09	0.04	0.13

Abbreviations: 5:2 MR, intermittent fasting plan consisting of 2 nonconsecutive fasting days and 5 days of habitual intake per week and meal replacement diet; BMI, body mass index (calculated as weight in kilograms divided by height in meters squared); HbA_1c_, hemoglobin A_1c_; HDL-C, high-density lipoprotein cholesterol; HOMA-IR, homeostasis model assessment of insulin resistance; LDL-C, low-density lipoprotein cholesterol.

SI conversion factors: To convert total cholesterol, HDL-C, and LDL-C to millimoles per liter, multiply by 0.0259; triglycerides to millimoles per liter, multiply by 0.0113; glucose to millimoles per liter, multiply by 0.0555; and HbA_1c_ to proportion of total hemoglobin, multiply by 0.01.

^a^ Only women (n = 106).

### Primary and Secondary Outcomes

At weeks 8 and 12, no patients in the 5:2 MR group required additional metformin for FPG level of 180.2 mg/dL or more and 2-hour plasma glucose of 250.5 mg/dL or more (to convert glucose to millimoles per liter, multiply by 0.0555). Only 1 patient in the metformin group had a FPG level of 218.0 mg/dL and consequently received additional empagliflozin.

**Figure 2.**
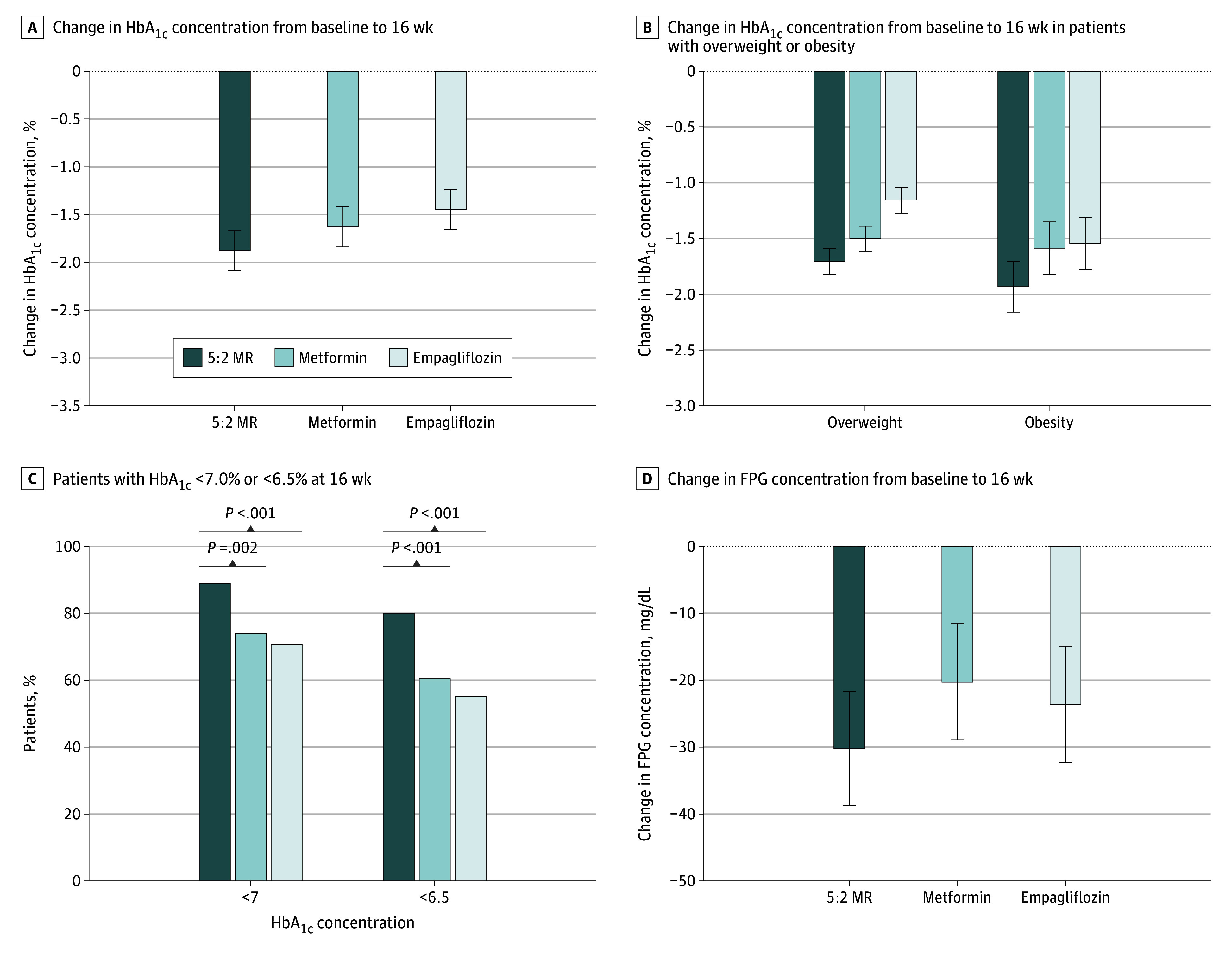
Glycemic Outcomes A, Changes in hemoglobin A_1c_ (HbA_1c_) concentration from baseline to 16 weeks. The adjusted least-squares mean (LSM) changes were intermittent fasting plan consisting of 2 nonconsecutive fasting days and 5 days of habitual intake per week and meal replacement diet (5:2 MR), −1.9% (SE, 0.2%); metformin, −1.6% (SE, 0.2%); and empagliflozin, −1.5% (SE, 0.2%) (to convert to proportion of total hemoglobin, multiply by 0.01). The adjusted LSM difference between 5:2 MR and metformin was −0.3% (95% CI, −0.5% to 0.1%), and the adjusted LSM difference between 5:2 MR and empagliflozin was −0.4% (95% CI, −0.6% to 0.2%). B, Changes in HbA_1c_ concentration from baseline to 16 weeks in patients with overweight or obesity. C, Percentage of patients with HbA_1c_ concentrations of less than 7.0% or of less than 6.5% at week 16. D, Changes in fasting plasma glucose (FPG) concentrations from baseline to 16 weeks (to convert glucose to millimoles per liter, multiply by 0.0555). Error bars display SEs.

**Figure 3.**
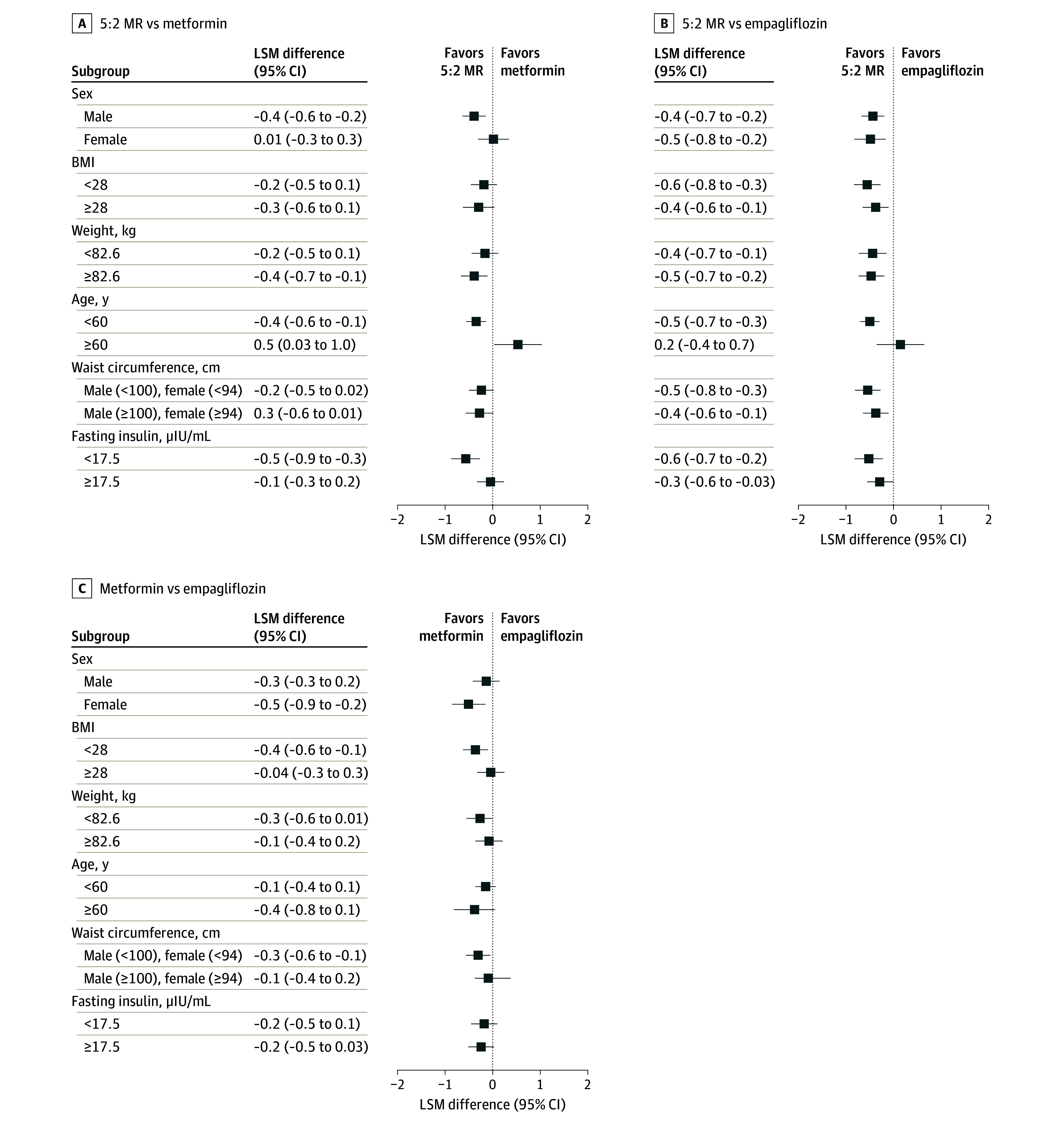
Post Hoc Subgroup Analysis of Hemoglobin A_1c_ (HbA_1c_) Concentrations at Week 16 by Intention-to-Treat Analysis Patients were randomized to receive intermittent fasting plan consisting of 2 nonconsecutive fasting days and 5 days of habitual intake per week and meal replacement diet (5:2 MR) (n = 135), metformin (n = 134), or empagliflozin (n = 136). BMI indicates body mass index (calculated as weight in kilograms divided by height in meters squared); and LSM, least-squares mean.

At week 16, patients in the 5:2 MR group showed the greatest reduction in HbA_1c_ level (LSM, −1.9% [SE, 0.2%]), significantly greater than patients receiving metformin (−1.6% [SE, 0.2%]; adjusted LSM difference, −0.3% [95% CI, −0.5% to −0.1%]) and empagliflozin (−1.5% [SE, 0.2%]; adjusted LSM difference, −0.4% [95% CI, −0.6% to −0.2%]) (Figure 2A; eTable 4 in Supplement 2). However, there was no difference between the 2 drug groups (adjusted LSM difference, –0.2% [95% CI, –0.4% to 0.01%]; *P* = .06). Post hoc subgroup analysis revealed that, apart from individuals aged 60 years or older, 5:2 MR mirrored the trend of HbA_1c_ reduction seen in the primary analysis (Figure 3). The unadjusted baseline characteristics of patients supported these findings (eTable 5 in Supplement 2). Similarly, analyses of patients who completed the 16-week treatment also yielded consistent results (eTable 6 in Supplement 2). Among individuals with obesity, 5:2 MR significantly reduced HbA_1c_ compared with metformin (LSM difference, −0.4% [95% CI, –0.6% to –0.1%]) and empagliflozin (LSM difference, −0.4% [95% CI, –0.7% to –0.1%]) (Figure 2B). More patients in the 5:2 MR group (88.9% [120 of 135]) achieved an HbA_1c_ level less than 7% compared with the metformin (73.9% [99 of 34]; *P* = .002) and empagliflozin (70.6% [96 of 136]; *P* < .001) groups (Figure 2C). Similarly, in the 5:2 MR group, 80.0% of patients (108 of 135) achieved an HbA_1c_ level of less than 6.5%, surpassing metformin (60.4% [81 of 134]; *P* < .001) and empagliflozin (55.1% [75 of 136]; *P* < .001). Fasting plasma glucose levels in the 5:2 MR group decreased by −30.3 mg/dL (95% CI, −46.7 to −13.7 mg/dL) (Figure 2D). At the end of 8-week follow-up, 72 of 94 participants (76.6%) in the 5:2 MR group maintained an HbA_1c_ less than 6.5% (eTable 7 in Supplement 2).

At week 16, patients in the 5:2 MR group showed greater weight loss (LSM, −9.7 kg [SE, 2.2 kg]) than those in the metformin group (−5.5 kg [SE, 2.3 kg]; adjusted LSM difference, −4.2 kg [95% CI, −6.2 to −2.2 kg]) and empagliflozin group (−5.8 kg [SE, 2.3 kg]; adjusted LSM difference, −3.9 kg [95% CI, −5.9 to −1.9 kg]; eFigure 3A and eTable 4 in Supplement 2), with a greater proportion of those in the 5:2 MR group achieving weight loss (eFigure 3B in Supplement 2). Subgroup analyses confirmed this trend (eFigure 4 in Supplement 2). In addition, patients in the 5:2 MR group had significant reduction in waist and hip circumference and systolic and diastolic blood pressure, but showed no notable differences in most metabolic markers, except for triglyceride and HDL-C, compared with patients receiving antidiabetic drugs (eTable 4 in Supplement 2).

### Safety

In the 5:2 MR group (n = 135), 1 patient experienced constipation, and 8 individuals (5.9%) had hypoglycemic symptoms, likely related to the low-energy diet (Table 2). In the metformin group (n = 134), 26 individuals (19.4%) had mild gastrointestinal symptoms, and 8 individuals (6.0%) had hypoglycemia. In the empagliflozin group (n = 136), 3 patients (2.2%) experienced urinary symptoms, 5 patients (3.7%) experienced hypoglycemia, and 1 patient reported thirst. Two patients in the empagliflozin group experienced serious adverse events, including severe rash and hospitalization due to increased blood ketones, which resolved with treatment.

**Table 2.** Adverse Events^a^

Adverse event	Patients, No. (%)		
	Metformin (n = 134)	Empagliflozin (n = 136)	5:2 MR (n = 135)
Any adverse events	36 (26.9)	11 (8.0)	9 (6.7)
Serious adverse events	0	2 (1.5)	0
Elevated blood ketones	0	1 (0.7)	0
Rash	0	1 (0.7)	0
Hypoglycemia	8 (6.0)	5 (3.7)	8 (5.9)
Gastrointestinal			
Nausea	6 (4.5)	0	0
Diarrhea	13 (9.7)	0	0
Abdominal pain	4 (3.0)	0	0
Constipation	0	0	1 (0.7)
Loss of appetite	3 (2.2)	0	0
Urologic	0	3 (2.2)	0
Other			
Headache	1 (0.7)	0	0
Fatigue	1 (0.7)	0	0
Thirstiness	0	1 (0.7)	0

Abbreviation: 5:2 MR, intermittent fasting plan consisting of 2 nonconsecutive fasting days and 5 days of habitual intake per week and meal replacement diet.

^a^ Data are from intention-to-treat dataset.

## Discussion

We found that among Chinese adults with overweight or obesity and newly diagnosed type 2 diabetes, the 5:2 MR approach achieved significant improvements in glycemic control and weight loss within a 16-week period, while also improving blood pressure and triglyceride and HDL-C levels. Therefore, 5:2 MR may potentially serve as an effective initial lifestyle intervention instead of antidiabetic drugs for early-stage type 2 diabetes.

Effective lifestyle interventions for patients with overweight and obesity and type 2 diabetes are crucial for achieving glycemic control and weight loss. Two single-center, small sample randomized clinical trials have confirmed that intermittent fasting can effectively reduce HbA_1c_ levels in these patients.^20,24^ The 5:2 intermittent fasting diet for 12 months resulted in a reduction of 0.5% in HbA_1c_ level compared with a continuous energy restriction diet, with no difference in weight loss.^20^ For patients with type 2 diabetes treated with insulin therapy, a 12-week 3:4 intermittent fasting intervention (3 days consuming 25% of recommended calories and 4 days without calorie restriction) led to a mean (SD) decrease of HbA_1c_ by 7.3 (12.0) mmol/mol (0.6% [1.1%]) and a mean (SD) weight loss of 4.8 (5.0) kg, with a daily total mean (SD) insulin dose reduction of 9 (10) IU.^24^ A recent systematic review reported that the changes in HbA_1c_ after intermittent fasting intervention ranged from −1.5% to −0.3%.^25^ Moreover, a meta-analysis of 2112 studies showed that partial or complete MR significantly reduced HbA_1c_ levels compared with conventional diabetes diets (−0.7% to −0.3%).^26^ Our results found that after a 16-week intervention with the 5:2 MR, the mean HbA_1c_ reduction was 1.9%, greater than those achieved with metformin (0.3%) and empagliflozin (0.4%). According to American Diabetes Association recommendations, individuals with an HbA_1c_ of less than 6.5% for at least 6 months after the initiation of lifestyle interventions are considered to achieve diabetes remission.^27^ In this study, 80.0% of patients reached this target with a 16-week 5:2 MR intervention. We acknowledge that the duration of our intervention was less than the recommended minimum of 6 months. Furthermore, at the end of the 8-week follow-up, 72 of 94 participants in the 5:2 MR group (76.6%) maintained an HbA_1c_ level of less than 6.5%, indicating that the 5:2 MR approach significantly and sustainably improves HbA_1c_ levels in patients with early type 2 diabetes.

In addition, our findings demonstrated that 5:2 MR reduced FPG levels, fasting insulin levels, C-peptide levels, and HOMA-IR. However, when compared with metformin and empagliflozin, the differences in fasting insulin levels, C-peptide levels, and HOMA-IR were not statistically significant. Animal studies have shown that fasting in diabetic mice can downregulate the expression of inflammatory factors, thereby alleviating inflammation.^28^ A 5:2 MR plan may reshape the gut microbiota, promote white adipose tissue browning, and consequently reduce insulin resistance and the occurrence of obesity.^29,30^ The MR used in this study contained omega-3 fatty acids and medium-chain fatty acids. Omega-3 fatty acids regulate leptin, inhibit fat synthesis, and promote fat breakdown.^31^ Medium-chain fatty acids reduce heterotopic fat, enhance brown fat thermogenesis, and increase insulin sensitivity.^32^

Compared with 2 antidiabetic drugs, 5:2 MR showed more significant and sustained benefits in weight loss and waist circumference reduction. Metformin exerts its effects by suppressing appetite, reducing insulin secretion, and improving gut microbiota.^33^ Sodium-glucose cotransporter-2 inhibitors directly reduce body weight by increasing glucose excretion in the kidneys.^34^ The DiRECT study confirmed that diabetes can be partially reversed through weight loss and proposed the “double cycle hypothesis,” suggesting that type 2 diabetes results from fat infiltration into the liver, pancreas, and muscle tissue, leading to the destruction of pancreatic β cells and tissue insulin resistance. Weight loss educes liver fat and significantly improves insulin resistance, and maintaining ideal body weight assists in β-cell function recovery, thus slowing down or even reversing the development of diabetes.^13,35^ Our study cannot conclusively determine whether the glycemic improvement in patients with type 2 diabetes is due to weight loss or the 5:2 MR approach itself, requiring further investigation. The 5:2 MR reduced blood pressure and total cholesterol and increased HDL-C levels, consistent with previous studies indicating improved metabolic parameters with intermittent fasting and MR,^26,32,36,37^ suggesting a potential cardiovascular protective effect.

The incidence of hypoglycemia was comparable across all 3 groups. When implementing a 5:2 MR intervention, it is essential to prevent hypoglycemia associated with low-energy diet. However, compared with medications, the 5:2 MR demonstrates favorable safety.

The 2020 China Guidelines emphasize lifestyle intervention as the foundational treatment for type 2 diabetes, with medication initiated only if lifestyle intervention fails to achieve glycemic control.^38^ The EARLY study, for the first time to our knowledge, directly compared 5:2 MR with 2 widely used antidiabetic medications, providing evidence for the 5:2 MR approach as an effective initial lifestyle intervention for Chinese patients with early-stage type 2 diabetes.

### Limitations

This study has some limitations. First, it enrolled only patients not taking antidiabetic medication with a baseline HbA_1c_ level of less than 9%, so the efficacy of 5:2 MR for those taking medication or with a greater baseline HbA_1c_ needs further validation. Second, the 3-month washout period for eligibility regarding antidiabetic agents, including insulin, was short. A longer period without medication use (6 or 12 months) could offer more insights into prior medications’ effects. Third, the 5:2 MR intervention’s short duration means its long-term efficacy, especially for newly diagnosed patients with type 2 diabetes and overweight or obesity remains to be confirmed. Long-term follow-up studies are under way to assess the durability of 5:2 MR.

## Conclusions

This randomized clinical study found that, for patients with newly diagnosed type 2 diabetes, a 16-week intervention with 5:2 MR could improve glycemic control and weight loss while also improving blood pressure, triglyceride levels, and HDL-C levels. Therefore, 5:2 MR may serve as an initial lifestyle intervention for patients with type 2 diabetes, providing an alternative to the use of metformin and empagliflozin medications.

## Notes

Supplement 1.
Trial Protocol

Supplement 2. **eMethods.** Multiple Imputation **eTable 1.** Study Centers, Location, Ethics Committee Approvals **eTable 2.** The Contents of Dietary and Exercise Guidance, and General Diabetes Education for All Participants **eTable 3.** Nutrition Facts of Kang Zhijun™ **eTable 4.** Changes in Primary and Secondary Outcomes From Baseline to Week 16 (Intention-to-Treat Analysis) **eTable 5.** Unadjusted Analyses of Outcomes From Baseline to Week 16 in the Three Groups **eTable 6.** Primary and Secondary Outcomes From Baseline to Week 16 in Three Groups (Completers Analysis) **eTable 7.** Primary and Secondary Outcomes From Baseline to Week 24 in Three Groups (Completers Analysis) **eFigure 1.** Study Design **eFigure 2.** The 5:2 MR Approach **eFigure 3.** Changes in Body Weight **eFigure 4.** Post-Hoc Subgroup Analysis of Weight Loss at Week 16 by Intention-to-Treat Analysis
eReference.

Supplement 3.
Data Sharing Statement
