# Supplementary material for: A 5:2 Intermittent Fasting Meal Replacement Diet and Glycemic Control for Adults With Diabetes: The EARLY Randomized Clinical Trial
Source: JAMA Netw Open. 2024 Jun 21;7(6):e2416786. doi: 10.1001/jamanetworkopen.2024.16786 (PMC11193124; doi:10.1001/jamanetworkopen.2024.16786)
Supplement: Supplement 1. — Trial Protocol [file jamanetwopen-e2416786-s001.pdf]

Study Protocol

Topic name: Exploration of Treatment Models for Newly Diagnosed Overweight/Obese Type 2 Diabetes Mellitus

Source: Beijing MetabolicControl Technology Co., Ltd

Applicant: Guo Lixin

Undertaking department: Department of Endocrinology, Beijing Hospital

I. Research background

Obesity and type 2 diabetes mellitus (T2DM) are common endocrine metabolic diseases and have become a worldwide epidemic. According to the data from IDF (International Diabetes Federation) in 2017, there were 425 million adults with diabetes worldwide, and it is estimated that by 2045, the number of people with diabetes may reach 629 million[1]. Epidemiological surveys in China show that the prevalence of adult diabetes is 11.6%, and China has the highest number of patients in the world. The proportion of overweight patients exceeds 40%, and the proportion of obese patients is about 25%[2]. Overweight and obesity not only promote the occurrence of diabetes but also are important risk factors affecting disease control in patients with T2DM. The Guidelines for the Prevention and Treatment of Type 2 Diabetes in China (2017 edition) recommend that the weight loss goal for overweight/obese patients is to reduce body weight by 5%~10% within 3~6 months. Positive lifestyle interventions that control weight not only reduce the risk of new onset diabetes in high-risk populations, but also significantly improve metabolic factors such as blood glucose, lipids, and blood pressure in patients with diabetes. Studies in Daqing, Finland, and the United States have shown that intensive lifestyle interventions can significantly reduce the risk of new onset diabetes by more than 50% by effectively reducing the weight of individuals with impaired glucose tolerance (IGT) [3,4]. The Look AHEAD study has also confirmed that intensive dietary and exercise interventions can effectively reduce weight and significantly improve fasting blood glucose, glycosylated hemoglobin, blood pressure, lipids, insulin resistance, and inflammation in obese patients with type 2 diabetes. It is important to note that intensive dietary and exercise interventions are often very difficult to maintain. The body may adapt to fasting, leading to increased appetite and reduced energy expenditure, preventing further weight loss or even weight rebound. Therefore, patients often cannot adhere to high-intensity exercise and low-calorie diets for a long time, resulting in failed weight control. Therefore, exploring a method that allows patients to persist for a long time without causing the body to adapt becomes the

direction of our research.

To improve adherence in weight loss patients while avoiding the adaptive response caused by fasting, intermittent fasting is an alternative to low-calorie diets. Intermittent fasting refers to regular periods of no or very limited calorie intake. Common patterns include daily fasting for 16 hours, alternate-day fasting, or weekly fasting for 2 non-consecutive days. During fasting periods, calorie intake is usually between 0-25% of calorie needs. On feeding days, dietary structure can be restricted without limiting the total calorie intake, and the total calorie intake can even reach 125% of normal needs[5]. Foreign studies have shown that intermittent fasting not only reduces the risk of diabetes in high-risk patients but also improves body weight and blood glucose control in patients with type 2 diabetes. Foreign experts have found that reducing food intake on non-fasting days by 9%-19% compared to normal requirements can lead to greater improvements in patient weight, fat mass, and blood glucose control. In addition, numerous human studies and animal experiments have confirmed that intermittent fasting also has benefits such as reducing oxidative stress and inflammation, enhancing autophagy and tissue repair ability, altering gut microbiota, and cardiovascular diseases [6-9].

However, there is currently a lack of evaluation studies on the effectiveness and safety of intermittent fasting for overweight and obese patients with type 2 diabetes in China. The intermittent fasting pattern used in this study will involve selecting two non-consecutive days per week (such as Monday and Thursday, or Tuesday and Friday, etc.) and replacing three meals with Kang zhijun™ Cereal Fruit Meal Bag No. 2 (A) (94 kcal/bag, 20g, 100g meal replacement bag A contains 17.2g protein, accounting for 29%, 19g fat, accounting for 32%, 57.6g carbohydrates, accounting for 19%, and sodium 368mg). For these two days, the diet plan is one egg for breakfast + meal replacement bag A, 100g low-sugar fruit (such as cucumber, tomato, orange, etc.) + meal replacement bag A for lunch, and 200-400g leafy greens (such as spinach, cabbage, etc.) + meal replacement bag A for dinner. For the remaining five days of the week, breakfast and lunch are normal diets (low oil and low sugar recommended), and

dinner is replaced with Anti-Chief Jun™ Cereal Fruit Meal Bag No. 5 (B) (88 kcal/bag, 20g, 100g meal replacement bag B contains 19.7 g protein, accounting for 33% of energy, 11.4 g fat, accounting for 19% of energy, 63.4g carbohydrates, accounting for 21% of energy, and sodium 349mg). This study aims to intervene in overweight and obese patients with newly diagnosed type 2 diabetes using an intermittent fasting pattern and follow up to evaluate the effectiveness and safety of intermittent fasting for weight and metabolic indicators in overweight and obese patients with newly diagnosed type 2 diabetes. To explore a safer and more effective dietary intervention mode for overweight and obese patients with newly diagnosed type 2 diabetes.

References

1. Cho NH, Shaw JE, Karuranga S, et al. IDF Diabetes Atlas: Global estimates of diabetes prevalence for 2017 and projections for 2045. Diabetes Res Clin Pract. 2018;138:271-281.
2. Xu Y, Wang L, He J, et al. Prevalence and control of diabetes in Chinese adults. JAMA. 2013;310(9):948-959.
3. Shen XX, Wang JP, Chen YY, et al. [Subjects with impaired glucose tolerance returned to normal glucose status for six years had lower long-term risk of diabetes: 20 years follow up of Daqing diabetes prevention study]. Zhonghua Nei Ke Za Zhi. 2019;58(5):372-376.
4. Baum A, Scarpa J, Bruzelius E, Tamler R, Basu S, Faghmous J. Targeting weight loss interventions to reduce cardiovascular complications of type 2 diabetes: a machine learning-based post-hoc analysis of heterogeneous treatment effects in the Look AHEAD trial. Lancet Diabetes Endocrinol. 2017;5(10):808-815.
5. Heilbronn LK, Smith SR, Martin CK, Anton SD, Ravussin E. Alternate-day fasting in nonobese subjects: effects on body weight, body composition, and energy metabolism. Am J Clin Nutr. 2005;81(1):69-73.
6. Li G, Xie C, Lu S, et al. Intermittent Fasting Promotes White Adipose Browning

and Decreases Obesity by Shaping the Gut Microbiota. Cell Metab. 2017;26(4):672-685 e674.

7. Lopez-Lluch G, Navas P. Calorie restriction as an intervention in ageing. J Physiol. 2016;594(8):2043-2060.

8. Qiu X, Brown K, Hirschey MD, Verdin E, Chen D. Calorie restriction reduces oxidative stress by SIRT3-mediated SOD2 activation. Cell Metab. 2010;12(6):662-667.

9. Malinowski B, Zalewska K, Wesierska A, et al. Intermittent Fasting in Cardiovascular Disorders-An Overview. Nutrients. 2019;11(3).

II. Research objectives:

To prospectively compare the effects of intermittent fasting meal replacement, metformin, and SGLT-2 inhibitor empagliflozin on blood glucose levels in overweight and obese patients with newly diagnosed type 2 diabetes. The secondary objectives of this study are to observe the proportion of patients with a reduction in glycosylated hemoglobin of greater than 0.4%, effectiveness (body weight/blood lipids/blood pressure/uric acid/islet function), compliance, and safety in the three treatment modes.

III. Research content:

A total of 405 overweight and obese patients with newly diagnosed type 2 diabetes who meet the inclusion and exclusion criteria will be selected and enrolled in the study after obtaining informed consent. The participants will be randomly divided into three groups: 5:2 MR group, metformin group, and empagliflozin group, in a ratio of 1:1:1. The intervention will last for 16 weeks, with follow-up visits every 4 weeks during the study period. Data on blood pressure, body weight, and body circumference will be recorded. Metabolic indicators such as blood glucose, blood lipids, uric acid, glycosylated hemoglobin, and insulin resistance index will be measured at baseline and at week 16. After week 16, all participants will no longer

receive intervention measures from this study, and whether to continue conventional hypoglycemic treatment will be determined by the researchers based on the patient's current condition. Telephone follow-up will be conducted at week 20 to collect data on blood pressure, body weight, body circumference, fasting and postprandial blood glucose levels. At week 24, changes in glycosylated hemoglobin, fasting blood glucose, blood lipids, urine routine, and body weight will be evaluated in all three groups.

IV. Research design and implementation plan

1. Study Subject Selection (Inclusion Criteria, Exclusion Criteria)

1) Inclusion Criteria: Participants must meet all of the following criteria to be eligible for inclusion: aged between 18 and 65 years old, diagnosed with type 2 diabetes according to the WHO (1999) diagnostic criteria, diabetes duration ≤1 year and no use of hypoglycemic drugs in the past 3 months, BMI≥25kg/m^2, HbA_{1C}≥9.0% and HbA_{1C}≥7.0%, stable weight (weight fluctuation within 5% in the previous 3 months), signed informed consent form and adhere to the study protocol.

2) Exclusion Criteria: If any of the following exclusion criteria are met, participants will not be included in this study. Diabetes other than clinically diagnosed type 2 diabetes; acute metabolic complications within 6 months prior to screening, including lactic acidosis, nonketotic hyperosmolar coma, diabetic ketoacidosis; severe acute or chronic complications of diabetes (such as myocardial infarction, stroke, congestive heart failure, blindness caused by diabetic retinopathy, renal failure, and severe foot ulcers); other endocrine diseases besides type 2 diabetes that are being treated and not controlled; hypoglycemia prone according to the investigator's judgment; taking weight-affecting medications (such as orlistat, glucocorticoids) or appetite and gastrointestinal motility-affecting medications in the past 3 months; severe liver function damage (ALT≥3ULN); recurrent urinary or reproductive system infections in the recent past; severe renal dysfunction (eGFR≤60ml/min/1.73m^2); heart failure

(NYHA III-IV stage); severe blood system diseases (such as thalassemia, iron deficiency anemia); alcohol consumption equivalent to >140 g/week of ethanol (>70 g/week for women); mental illness unable to cooperate with the trial; unable to eat by mouth; history of gastrointestinal surgery; intolerant or allergic to meal replacement component; allergic to metformin or empagliflozin; pregnant and breastfeeding women; history of tumors within the past 5 years.

2. Study Design (Research Type, Control Method, Randomization and Blinding Level)

This study is a prospective intervention study, where the participants will be randomly assigned to the intermittent fasting group, metformin group, and empagliflozin group after enrollment, with a 1:1:1 ratio. The intermittent fasting group serves as the intervention group, while the metformin group and empagliflozin group serve as control groups. No blinding will be applied among the three groups.

3. Research Process and Follow-up Plan

3.1 Baseline Period: The measurement of patients' vital signs and body circumference indicators includes blood pressure, height, weight, waist circumference, hip circumference, etc. In the fasting state, the blood routine, liver and kidney function, glucose, lipids, uric acid and other biochemical indicators, glycosylated hemoglobin will be detected. The standard meal will be used to evaluate the insulin function and insulin resistance status of the subjects. Urine routine examination will also be performed. Patients will be randomly assigned to the intermittent fasting group, metformin group, and empagliflozin group according to a 1:1:1 ratio.

- **5:2 MR Group:** A meal replacement package will be provided. The specific plan is to select two non-consecutive days within a week (such as Monday and Thursday, or Tuesday and Friday, etc.) to replace three meals with Anti-Diet King™ Cereal Fruit Vegetable Meal Package No. 2 (A) (94kcal/package 20g, 100g meal replacement package A contains protein 17.2g accounting for 29%, fat 19g accounting for 32%, carbohydrates 57.6g accounting for 19%, sodium 368mg). For these two days, the dietary plan is breakfast of one egg + meal

replacement package A, lunch of 100g low-sugar fruits (such as cucumber, tomato, orange, etc.) + meal replacement package A, and dinner of 200-400g leafy greens (such as spinach, cabbage, etc.) + meal replacement package A. For the remaining five days of the week, breakfast and lunch are normal diets (low oil and sugar recommended), and dinner is replaced with Anti-Diet King™ Cereal Fruit Vegetable Meal Package No. 5 (B) (88kcal/package 20g, 100g meal replacement package B contains protein 19.7 g accounting for 33%, fat 11.4 g accounting for 19%, carbohydrates 63.4g accounting for 21%, sodium 349mg).

- **Metformin Group:** The starting dose is 0.5g per time, twice a day, and gradually increased to the maximum tolerated dose of 0.5g per time, 2 to 4 times a day (1g-2g/day).
- **Empagliflozin Group:** Empagliflozin will be given once daily, 10mg per time.

The researchers will provide glucose recording diaries and dietary exercise recording diaries to the participants in all three groups.

3.2 Intervention Period：

- **Follow-up at Week 4, Week 8, and Week 12:** The participants' blood glucose record books and dietary exercise diaries will be collected. The physician will assess the participants' blood glucose status and measure their vital signs and body circumference indicators, including blood pressure, height, weight, waist circumference, hip circumference, etc., and record any medication side effects such as gastrointestinal reactions, urinary/reproductive system infections, hypoglycemia, etc. The researcher will provide new meal replacement packages or medications and distribute blood glucose record books and dietary diaries.
- **Follow-up at Week 16**: The participants' blood glucose record books and dietary exercise diaries will be collected again. The physician will assess the participants' blood glucose status and measure their vital signs and body circumference indicators, including blood pressure, height, weight, waist circumference, hip circumference, etc., and record any medication side effects such as gastrointestinal reactions, urinary/reproductive system infections, hypoglycemia,

etc. The researcher will provide new meal replacement packages or medications and distribute blood glucose record books and dietary diaries. The participants will undergo fasting blood tests for liver and kidney function, blood glucose, lipids, uric acid, glycosylated hemoglobin, and urine routine examination to evaluate their pancreatic function and insulin resistance status with a standard meal.

3.3 Follow-up Period (Week 16 to Week 24): After Week 16, all participants will no longer receive the intervention measures of this study. Whether to continue with regular blood sugar lowering treatment will be determined by the researcher based on the patient's current condition. The researcher will conduct a telephone visit at Week 20 to record the participants' weight, waist circumference, hip circumference, blood pressure, blood glucose, and other indicators. At Week 24, all three groups of participants will return to the hospital for a follow-up visit. The physician will assess the participants' blood glucose status and measure their vital signs and body circumference indicators, including blood pressure, height, weight, waist circumference, hip circumference, etc. Fasting blood tests will be conducted to evaluate liver and kidney function, blood glucose, lipids, uric acid, glycosylated hemoglobin, and urine routine examination.

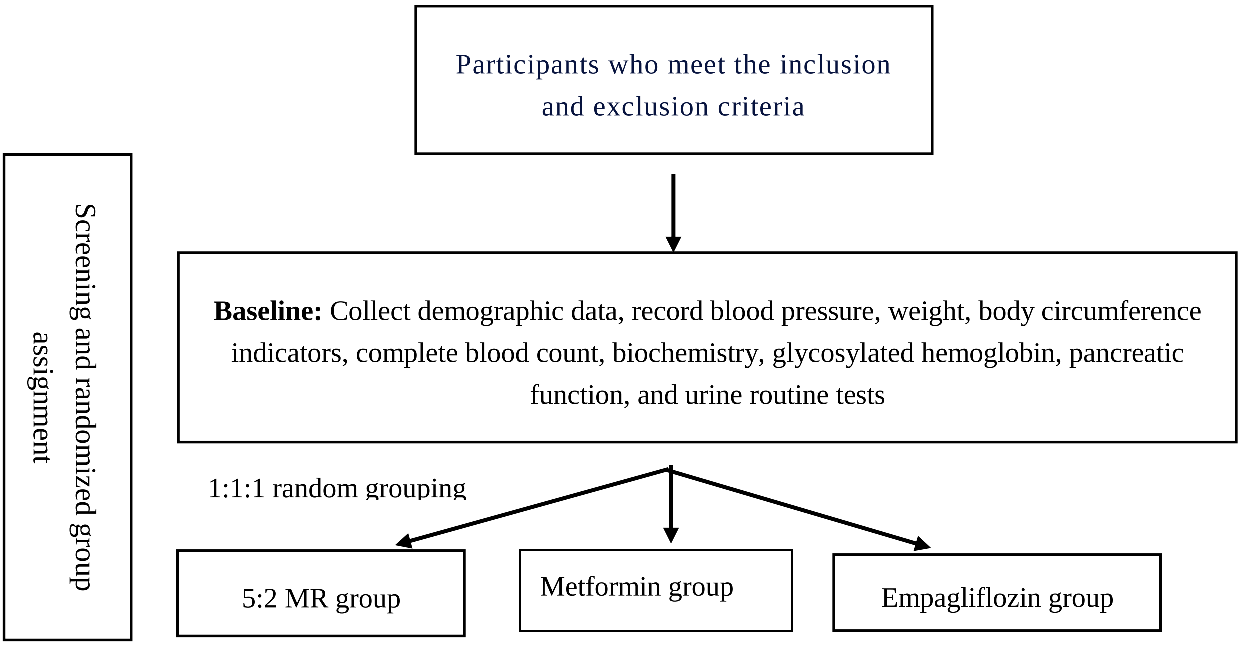

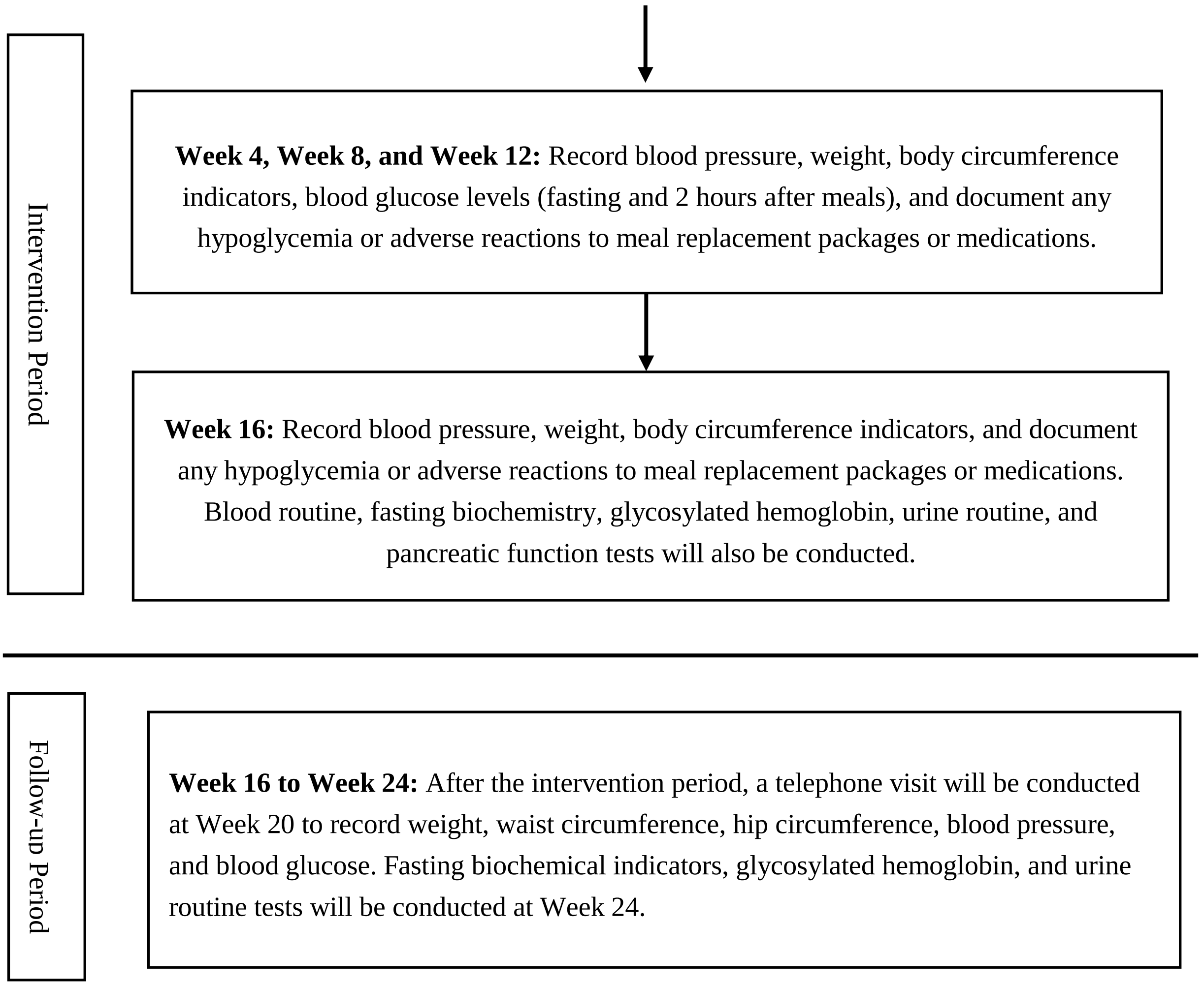

4. Sample size calculation and statistical analysis methods

Sample size calculation: The main purpose of this study is to compare the decline in HbA1C from baseline among the three interventions. According to the literature review, the parameters set are α=0.05, β=0.19, the minimum acceptable difference is 0.1, the standard deviation is 0.15, and the multiple paired comparison test for quantitative data, namely the Turkey-Kramer method, will be used to calculate the sample size for the three groups. Using PASS15 software, each group requires 108 participants, a total of 324 participants. Considering a 20% dropout rate, a total of 405 participants will be needed for this study.

Statistical methods: Descriptive statistical analysis methods will be used, including

calculating the mean, standard deviation, median, minimum and maximum values for continuous variables; calculating frequency and percentage for categorical variables. The differences between the experimental group and each control group will be compared using chi-square or Fisher's exact test for categorical variables, and ANOVA or covariance analysis for continuous variables. If the data distribution does not meet the assumptions of hypothesis testing, non-parametric methods will be used. Unless otherwise specified, the statistical significance level is set at 0.05.

5. Research evaluation indicators (methods for evaluating parameters, endpoint events, recording and analysis)

1) Main evaluation indicators: Evaluate the amount and degree of decrease in HbA_{1c} levels from baseline in the three study groups at Week 16.

2) Secondary evaluation indicators: Evaluate the proportion of participants in each group who have achieved weight loss of ≥5% at Week 16. Additionally, evaluate the changes in body weight, blood pressure, waist circumference, hip circumference, fasting blood glucose, 2-hour postprandial blood glucose, systolic blood pressure, lipids, uric acid, and insulin resistance status (HOMA-IR index) in the three groups at week 16.

6. Other (withdrawal criteria for participants, criteria for discontinuing the study, concomitant medication and handling of adverse events, etc.)

1) Withdrawal criteria: Participants can decide to withdraw from the study at any time for any reason, or their withdrawal may be decided by the researcher. Every effort should be made to record the reason for discontinuation of medication and record it in the CRF/eCRF. Reasons for termination of intervention include: significant intolerance to intermittent fasting or empagliflozin medication (AE); use of prohibited concomitant medications; other poor compliance and/or major protocol deviations; participant's desire to withdraw from the study; pregnancy or planning to become pregnant; ceasing contraception; frequent hyperglycemia or hypoglycemia, according to the researcher's judgment, the study no longer provides a positive benefit/risk ratio for the participant, and the participant needs to switch to a diabetes

treatment plan other than this study. Before deciding to permanently terminate the participant's study intervention treatment, any laboratory abnormal values should be immediately rechecked for confirmation.

2) Discontinuing Criteria: The ethics review committee or regulatory authority of the research center may terminate the study at the research center for medical, safety, regulatory, or other reasons that are consistent with applicable laws, regulations, and GCP. If the study is terminated or interrupted prematurely, the researcher will collect and retain all study records and complete as much of the CRF as possible.

3) Handling of adverse events: Intermittent fasting meal replacement programs may cause physical symptoms such as gastrointestinal discomfort, hunger, fatigue, headache, dizziness, hypoglycemia, and mild cognitive adverse reactions including lack of concentration. Gastrointestinal discomfort includes symptoms such as abdominal distension, abdominal pain, diarrhea, and constipation. It is recommended that participants in the intermittent fasting group ensure adequate daily water intake of at least 1500-2000ml/day. If a participant has mild discomfort that can be tolerated and does not require special treatment, no action is needed. If a participant has moderate discomfort, symptomatic treatment can be given; if severe discomfort occurs, the study should be terminated.

Common adverse reactions to metformin include diarrhea, nausea, vomiting, abdominal bloating, fatigue, indigestion, abdominal discomfort, and headache. Other less common adverse reactions include abnormal stools, hypoglycemia, myalgia, dizziness, nail abnormalities, rashes, increased sweating, taste abnormalities, chest discomfort, chills, flu-like symptoms, hot flashes, palpitations, and weight loss. Metformin can reduce vitamin B_{12} absorption but rarely causes anemia. Rare cases of lactic acidosis occur within the therapeutic dose range of metformin. If a participant has significant gastrointestinal reactions, the dose of metformin can be reduced until the gastrointestinal tract adapts and then increased to the maximum tolerated dose.

The main adverse reactions of empagliflozin include urinary tract infections, reproductive system infections, increased urination, dysuria, blood pressure decrease,

elevated serum creatinine levels, rhinitis, constipation, headaches, lower extremity pain, etc. Prior to medication, participants should be informed to increase their water intake appropriately to reduce the risk of dysuria and urinary/reproductive system infections. If a participant experiences dysuria, they should undergo immediate urine routine examination. If urinary tract infection occurs, appropriate anti-infective drugs can be used. If the patient can tolerate it, medication can continue; if not, the study should be terminated.

4) Remedial measures during treatment: If fasting blood glucose ≥ 10mmol/L and 2-hour postprandial blood glucose ≥13.9mmol/L are detected at the 8th and 12th week follow-up visits, participants in the metformin group should add empagliflozin, those in the empagliflozin group should add metformin, and those in the intermittent fasting group should add metformin. If fasting blood glucose ≥ 13.9mmol/L and 2-hour postprandial blood glucose ≥16.7mmol/L are still detected on a retest day, participants need to withdraw from the study.

V. Data Management Plan

1.Original Data Management

1) Researchers must ensure that the data is true, complete, and accurate;

2) All items in the trial record must be filled in without any empty or missing items (blank spaces can be crossed out). Any corrections can only be made by crossing out the original text, adding a note with the revised data and the reason for the correction, and signing and dating it by the researcher. The original record cannot be erased or covered.

3) The laboratory examination items should be complete.

2.Data recording

The data related to the participants on the case report form should be recorded using the participant coding method. Participants can only be identified by their participant coding or the initials of their name.

Data entry: The primary researcher needs to promptly and accurately enter the data

from the original record form into the CRF. The CRF is not considered as the original record, and its content originates from the original case report. Another researcher should verify the consistency of the CRF data with the original record form, laboratory test results, etc. If any issues are found, they can communicate with the primary researcher at any time. The CRF will be collected and summarized by a designated person and sent to the Department of Endocrinology at Beijing Hospital. After checking and cleaning the questionnaires, a designated person will double-enter the data into the Epidata database and perform consistency checks. The CRF will be kept in a locked room under specified conditions, and non-researchers cannot access it. The electronic database will be stored on a computer with a password, and non-researchers will not have access permissions. After the study is completed, the CRF will be archived by the research institution.

Researchers must retain the original documents of each participant who participates in the study. All information on the case report form must come from these original documents, which should include all demographic information and treatment information, including laboratory examination data, as well as signed informed consent forms indicating the research number and title.

3. Quality Control

(1) The research institution must be a clinical research base with clinical research qualifications determined by the state.

(2) Researchers must be physicians who have undergone clinical training and work under the guidance of senior professionals. Standardized operating procedures are used in the study to ensure the implementation of quality control and quality assurance systems for clinical research.

(3) All observed results and abnormal findings during clinical research should be carefully verified and recorded in a timely manner to ensure data reliability. The recording and transfer of clinical data must be carried out by experienced physicians, supervised or checked by designated personnel, to ensure the scientificity and accuracy of the data.

(4) All conclusions from clinical research must be derived from original data. The responsible physician should complete, detail, and accurately fill in the CRF. After each patient is registered, the CRF should be completed as soon as possible and submitted to the superior physician for signature before being submitted or kept in accordance with the specified procedures.

(5) All data related to the study should be centrally managed and analyzed. Procedures for data storage, data transfer, and data retrieval should be established. The stored data includes the CRF, subject code list, and various original medical documents. The transferred data includes the CRF and summary data required for analysis.

(6) Supervisors and relevant hospital leaders have the right to access trial-related information and original records after obtaining consent from the principal investigator. When finalizing the entire trial report, the lead unit and primary researchers can access or review all participant data and original records after obtaining consent.

(7) When summarizing and analyzing the results of clinical trials, standard statistical analysis methods must be used and professional statisticians should be consulted. To ensure the reliability and completeness of trial data, the principal investigator conducts regular systematic monitoring of the clinical trial site to determine if the execution complies with the research protocol and if the reported data is consistent with the records of the participating units. Each monitoring visit and interview should be documented in a visit report.

VI. Ethical issues

This study will strictly follow the Declaration of Helsinki and relevant laws and regulations for ethical review in our country, and will conduct research in strict accordance with the research plan. Before the start of the study, relevant information must be submitted to the hospital ethics committee for review and approval before the study can proceed. Participants must sign an informed consent form before

participating in the study. During the study, any modifications to the research plan, informed consent form, etc. must be resubmitted to the hospital ethics committee for review and approval before implementation. When publishing research information and data obtained from this study at scientific conferences or in scientific journals, the identity of participants will not be disclosed.

(1) Protective Plan for Participants

1) After the establishment of the research project, it must first undergo ethical review and can only proceed after approval.

2) Subjects will be selected based on the order of diagnosis, ensuring equal opportunities for each subject to participate in the study and minimizing selection bias.

3) Before the start of the study, the investigator will introduce the research purpose, content, schedule, benefits, risks, etc. in a language that the subjects can understand and comprehend.

4) Adequate time will be given for subjects to read or consider the contents of the informed consent form. If there are any questions, they should be raised in a timely manner. The investigator should carefully explain any questions raised by the subjects, emphasizing that their participation is entirely voluntary and they can withdraw from the study at any time without any consequences.

5) If a subject agrees to participate in the study, they should sign or fingerprint on the informed consent form. The investigator should also sign their name and date. If a subject is unable to sign or fingerprint, a witness should be present and sign and date, certifying that the investigator has provided truthful and complete information about the informed consent form to the subject according to its contents, and that the subject has voluntarily participated in this study.

6) Upon signing the informed consent form, it indicates that the subject has voluntarily participated in this study. After verification, the investigator will retain the original informed consent form while the subject may keep a copy.

(2) Ethical Report Plan

This study will adhere to the Declaration of Helsinki and Good Clinical Practice (GCP) guidelines, as well as other domestic and international laws and regulations. In order to protect participants and meet ethical requirements, this study will be conducted in strict accordance with the following conditions:

1) Prior to the initiation of the study, it must first undergo ethical review and approval before any research can proceed.

2) If there are any changes to the principal investigator or modifications to the clinical trial/research protocol, informed consent form (if applicable), recruitment materials (if applicable), etc. during the course of the study, an amendment application must be submitted for review and approval by the ethics committee before implementation.

3) If serious adverse events occur in study participants during the course of the study, the applicant must promptly submit a serious adverse event report to the ethics committee.

4) If participants who do not meet the inclusion criteria or meet the exclusion criteria are included in the study, if participants are not withdrawn from the trial/study despite meeting the criteria for discontinuation, if incorrect treatment or dosages are administered, if concomitant medications prohibited by the protocol are given, or if the trial/study is not conducted in accordance with the protocol, which may have a negative impact on the rights/health of participants or the scientific integrity of the trial/study, deviating from GCP principles; in such cases, the researcher must submit a deviation report to the ethics committee.

5) Regular progress reports must be submitted to the ethics committee, and a final report must be submitted upon completion of the study.
