# Supplementary material for: A 5:2 Intermittent Fasting Meal Replacement Diet and Glycemic Control for Adults With Diabetes: The EARLY Randomized Clinical Trial
Source: JAMA Netw Open. 2024 Jun 21;7(6):e2416786. doi: 10.1001/jamanetworkopen.2024.16786 (PMC11193124; doi:10.1001/jamanetworkopen.2024.16786)
Supplement: Supplement 2. — eMethods. Multiple Imputation eTable 1. Study Centers, Location, Ethics Committee Approvals eTable 2. The Contents of Dietary and Exercise Guidance, and General Diabetes Education for All Participants eTable 3. Nutrition Facts of Kang Zhijun™ eTable 4. Changes in Primary and Secondary Outcomes From Baseline to Week 16 (Intention-to-Treat Analysis) eTable 5. Unadjusted Analyses of Outcomes From Baseline to Week 16 in the Three Groups eTable 6. Primary and Secondary Outcomes From Baseline to Week 16 in Three Groups (Completers Analysis) eTable 7. Primary and Secondary Outcomes From Baseline to Week 24 in Three Groups (Completers Analysis) eFigure 1. Study Design eFigure 2. The 5:2 MR Approach eFigure 3. Changes in Body Weight eFigure 4. Post-Hoc Subgroup Analysis of Weight Loss at Week 16 by Intention-to-Treat Analysis eReference. [file jamanetwopen-e2416786-s002.pdf]

Supplementary Online Content

Guo L, Xi Y, Jin W, et al. A 5:2 intermittent fasting meal replacement diet and glycemic control for adults with diabetes: the EARLY randomized clinical trial. *JAMA Netw Open.* 2024;7(6):e2416786. doi:10.1001/jamanetworkopen.2024.16786

eMethods. Multiple Imputation
eTable 1. Study Centers, Location, Ethics Committee Approvals
eTable 2. The Contents of Dietary and Exercise Guidance, and General Diabetes Education for All Participants
eTable 3. Nutrition Facts of Kang Zhijun™
eTable 4. Changes in Primary and Secondary Outcomes From Baseline to Week 16 (Intention-to-Treat Analysis)
eTable 5. Unadjusted Analyses of Outcomes From Baseline to Week 16 in the Three Groups
eTable 6. Primary and Secondary Outcomes From Baseline to Week 16 in Three Groups (Completers Analysis)
eTable 7. Primary and Secondary Outcomes From Baseline to Week 24 in Three Groups (Completers Analysis)
eFigure 1. Study Design
eFigure 2. The 5:2 MR Approach
eFigure 3. Changes in Body Weight
eFigure 4. Post-Hoc Subgroup Analysis of Weight Loss at Week 16 by Intention-to-Treat Analysis
eReference.

This supplementary material has been provided by the authors to give readers additional information about their work.

© 2024 Guo L et al. *JAMA Network Open.*

eMethods. Multiple Imputation

In this study, multiple imputation was employed under the assumption of "missing at random" (MAR). This means that the probability of data being missing is unrelated to the missing values themselves but is only related to the observed data. For the imputation of continuous variables, we utilized a linear regression model. In this approach, each variable with missing data was modeled as a function of the other variables in the dataset. The linear regression model was used to estimate the relationships between the variables, and these relationships were then used to predict the missing values. Specifically, the missing value for a particular variable was estimated as a linear combination of the observed values of the other variables, with the coefficients determined by the regression model. The process of multiple imputation involves creating several imputed datasets, each with slightly different imputed values. This is done to account for the uncertainty in the imputation process. The analysis is then performed on each of these datasets separately, and the results are combined to produce a single set of estimates that reflect the variability due to missing data.

© 2024 Guo L et al. *JAMA Network Open.*

eTable 1. Study Centers, Location, Ethics Committee Approvals

Center	Location (city)	Ethical Approval Number	Acceptance Date (mm-dd-yy)
Beijing Hospital	Beijing	2020BJYYEC-220-02	11/13/2020
Hebei Provincial People's Hospital	Shijiazhuang	Research Ethics Review No. 60 (2021)	04/26/2021
The Second Hospital of Hebei Medical University	Shijiazhuang	2021-C004	04/08/2021
Henan Provincial People's Hospital	Zhengzhou	Research Ethics Review No. 86 (2021)	06/28/2021
The First Affiliated Hospital of Zhengzhou University	Zhengzhou	TA2021-227-002	08/12/2021
Nanyang Central Hospital	Nanyang	2021-009-01	05/29/2021
Sir Run Run Hospital, Nanjing Medical University	Nanjing	2021-IIT-001	04/06/2021
The First Affiliated Hospital of Soochow University, Soochow	Soochow	2020BJYYEC-220-02	05/05/2021
The Third Affiliated Hospital of Jinzhou Medical University	Jinzhou	JYDSY-YP-IEC-2021-001-1	05/06/2021

© 2024 Guo L et al. *JAMA Network Open.*

eTable 2. The Contents of Dietary and Exercise Guidance, and General Diabetes Education for All Participants

<table>
<tr><th>Item</th><th>Contents[1]</th></tr>
<tr><td>General Diabetes Education</td><td>Diabetes overview, chronic complications and comorbidities of diabetes, acute complications of diabetes, psychological stress and coping in diabetes patients.</td></tr>
<tr><td>Dietary Guidance</td><td>I. Dietary planning

Calculation of Ideal Weight Ideal weight (kg) =Height (cm) -105. Within ± 10% of this value, it is within the normal range.
Based on ideal weight and participation in physical labor, the total daily calorie intake from food can be calculated. Daily total calorie requirement=ideal weight × The amount of calories required per kilogram of body weight.
Energy demand table for different physical labor

<table>
<tr><th rowspan="2">Physical Activity Level</th><th colspan="3">Calories/Ideal Body Weight per Day</th></tr>
<tr><th>Lean</th><th>Normal Weight</th><th>Obesity</th></tr>
<tr><td>Bed Rest</td><td>20-25</td><td>15-20</td><td>15</td></tr>
<tr><td>Light Physical Activity</td><td>35</td><td>30</td><td>20-25</td></tr>
<tr><td>Moderate Physical Activity</td><td>40</td><td>35</td><td>30</td></tr>
<tr><td>Heavy Physical Activity</td><td>45</td><td>40</td><td>35</td></tr>
</table>

Distribution of the three nutrients
</td></tr>
</table>

© 2024 Guo L et al. *JAMA Network Open.*

	Recommended Energy Proportions for Daily Diet (%)	Source
Carbohydrates	50%~60%	Cereals, tubers, legumes, etc.
Protein	15%~20%	Animal protein (various lean meats, fish, shrimp, etc.)
		Plant protein (soybeans and their products, cereals)
Fats	≤30%	Saturated fatty acids, polyunsaturated fatty acids, monounsaturated fatty acids

II. Reasonable arrangement of meals

Diabetes patients should have at least three meals a day, so that staple food and protein are evenly distributed in the three meals, and they should be rationed regularly, generally according to 1/5, 2/5 or 1/3. For those who are prone to hypoglycemia, a small portion of staple food can be evenly distributed in the main meal as an additional meal between the two main meals. In addition to the main course, protein foods such as milk, eggs, and dried tofu can be added to the bedtime meal.

III. Restricting alcohol consumption

IV. Scientific selection of fruits

When the fasting blood glucose is controlled below 7.0mmol/L (126mg/dl), the postprandial blood glucose is less than 10mmol/L (180mg/dl), the glycated hemoglobin is less than 7.5%, and there is no significant fluctuation in blood glucose, fruits can be chosen. It is best to eat between two meals. Those who are not satisfied with disease control should not eat it temporarily. They can eat a small amount of raw cucumbers and tomatoes.

	**V. Precautions for dietary therapy**<br>• Carbohydrates Sweet potatoes, potatoes, yams, taro, lotus roots and other root vegetables have a high starch content and cannot be eaten casually. They need to be exchanged with grain. Strictly limit the intake of white sugar, brown sugar, honey, jam, chocolate, various sweets, sugary drinks, ice cream and various sweet Dim sum.<br>• Fat and cholesterol diabetes patients should eat less fried food. Nuts are high in fat content and should be consumed less. The daily intake of cholesterol should be less than 300 milligrams.<br>• Vitamin and mineral diabetes patients can eat more fresh vegetables, and try to eat raw as much as possible to ensure the full absorption of vitamin C and other nutrients. The intake of salt should be limited to 6 grams per day.
**Exercise Guidance**	**I. Preparation before exercise**<br>Before starting any exercise plan, patients undergoing a comprehensive physical examination should thoroughly screen for any potential complications, exclude potential diseases or injuries, and exclude risk factors to ensure exercise safety. Make exercise plans and discuss with doctors or full-time diabetes educators whether their physical conditions are suitable for exercise, and determine the exercise mode and amount. If blood glucose is less than 5.6 mmol/L, additional carbohydrates should be consumed before exercising.<br>**II.** Exercise style, intensity, time, and frequency<br>The exercise intensity selected by diabetes patients should be 60~70% of the maximum exercise intensity. The intensity of exercise can also be controlled based on one's feelings, that is, the whole body is hot and sweating, but not sweating

© 2024 Guo L et al. *JAMA Network Open.*

	profusely. Patients with diabetes can choose moderate and low-intensity aerobic exercise. Exercise should be carried out about 1-2 hours after the start of a meal because blood glucose is high at this time and low blood glucose is less likely to occur during exercise. The duration of each exercise is approximately 30-60 minutes. The frequency of exercise diabetes patients should adhere to at least 3 to 4 times of moderate and low-intensity exercise every week.

© 2024 Guo L et al. *JAMA Network Open.*

eTable 3. Nutrition Facts of Kang Zhijun™

	Package A	Package B
Calories	94kcal per serving	88kcal per serving
Weight	20g per serving	20g per serving
Protein	17.2g per100g; 29%	19.7g per 100g; 33%
Fat	19g per 100g; 32%	11.4g per 100g; 19%
Total carbohydrates	57.6g per 100g; 19%	63.4g per 100g; 21%
Sodium	368mg per 100g	349mg per 100g
Ingredients	Medium-chain triglycerides, extra virgin olive oil, sea buckthorn oil, DHA algal oil, maltodextrin, fructo-oligosaccharides, whole milk powder, soy protein powder, inulin, xylitol, sweet orange powder, cheese powder, calcium (calcium carbonate), iron (ferric pyrophosphate), zinc (zinc oxide), vitamin A, vitamin D, vitamin E, vitamin B1, vitamin B2, vitamin B6, vitamin B12, vitamin C, nicotinic acid, folic acid, food additives (casein	Grain flour (rice, soybeans, beer malt, oats, brown rice), xylitol, inulin, soy protein isolate, resistant dextrin, conjugated linoleic acid type edible vegetable oil, whey protein, kudzu powder, Chinese yam powder, goji berry powder, white kidney bean powder, food additives (casein sodium, monoglyceride and diglyceride fatty acid esters, sucralose)

	sodium, monoglyceride and diglyceride fatty acid esters, sucralose)

[a] One serving of Kang zhijun™ A (20g, 94 kcal, containing 11.5g carbohydrates, 3.4g protein, and 3.8g fat) was dissolved in 200ml of water to replace breakfast, lunch, and dinner.

[b] For the remaining five days of the week, patients followed their regular diet for breakfast and lunch, and used one serving of Kang zhijun™ B (20g dissolved in 200ml of water) to replace dinner. Each serving of meal powder B provided 88 kcal with 12.7g of carbohydrates, 3.9g of protein, and 2.3g of fat.

© 2024 Guo L et al. *JAMA Network Open.*

eTable 4. Changes in Primary and Secondary Outcomes from Baseline to Week 16 (Intention-to-Treat Analysis)[a]

	**Metformin (n=134)**	**Empagliflozin (n=136)**	**5:2 MR (n=135)**	**Treatment Comparison** LSM difference (SE); 95% CI; *P* value		
				5:2 MR *vs* Metformin	5:2 MR *vs* Empagliflozin	Metformin *vs* Empagliflozin
**Primary endpoint**						
HbA_{1c}, %	-1.63 (0.21)	-1.45 (0.21)	-1.88 (0.21)	-0.25 (0.10) (-0.45, -0.06) .011	-0.43 (0.10) (-0.63, -0.24) .0001	-0.18 (0.10) (-0.37, 0.01) .061
**Secondary endpoints**						
Bodyweight, kg	-5.5 (2.3)	-5.8 (2.3)	-9.7 (2.2)	-4.2 (1.0) (-6.2, -2.2) <.0001	-3.9 (1.0) (-5.9, -1.9) <.0001	0.3 (1.0) (-1.7, 2.3) .767
Body mass index, kg/m^2	-1.82 (0.79)	-1.96 (0.79)	-3.35 (0.76)	-1.53 (0.35) (-2.23, -0.84) <.0001	-1.39 (0.35) (-2.08, -0.70) <.0001	0.14 (0.35) (-0.54, 0.82) .681
Fasting plasma glucose, mmol/L	-1.12 (0.48)	-1.31 (0.48)	-1.68 (0.47)	-0.56 (0.22) (-1.00, -0.12) .012	-0.37 (0.22) (-0.80, 0.07) .097	0.19 (0.22) (-0.24, 0.62) .376
Fasting insulin, μU/mL	-3.78 (8.17)	-4.58 (8.14)	-10.69 (7.89)	-6.90 (3.78) (-14.34, 0.53) .069	-6.10 (3.77) (-13.51, 1.30) .106	0.80 (3.70) (-6.48, 8.08) .829
Fasting C-peptide,	-0.28 (0.36)	-0.35 (0.36)	-0.6 (0.35)	-0.32 (0.17)	-0.25 (0.17)	0.07 (0.16)

© 2024 Guo L et al. *JAMA Network Open.*

ng/mL				(-0.65, 0.01)<br>.058	(-0.58, 0.08)<br>.134	(-0.25, 0.39)<br>.676
HOMA-IR	-3.02 (4.22)	-3.23 (4.21)	-5.33 (4.08)	-2.30 (1.95)<br>(-6.14, 1.54)<br>.239	-2.09 (1.95)<br>(-5.92, 1.73)<br>.283	0.21 (1.91)<br>(-3.55, 3.97)<br>.913
Waist circumference, cm	-4.9 (2.4)	-4.6 (2.3)	-9.9 (2.3)	-5.0 (1.1)<br>(-7.0, -2.9)<br><.0001	-5.2 (1.1)<br>(-7.3, -3.2)<br><.0001	-0.3 (1.0)<br>(-2.3, 1.8)<br>.802
Hip circumference, cm	-3.5 (2.1)	-3.4 (2.1)	-5.5 (2.1)	-2 (1.0)<br>(-3.9, -0.1)<br>.038	-2.1 (1.0)<br>(-4.0, -0.2)<br>.031	-0.1 (0.9)<br>(-1.9, 1.8)<br>.94
Waist-to-hip ratio	-0.03 (0.05)	-0.02 (0.05)	-0.06 (0.04)	-0.03 (0.02)<br>(-0.07, 0.01)<br>.106	-0.04 (0.02)<br>(-0.08, 0.00)<br>.049	-0.01 (0.02)<br>(-0.05, 0.03)<br>.721
Systolic blood pressure, mm Hg	-1.6 (3.2)	-2.1 (3.2)	-6.7 (3.1)	-5.0 (1.5)<br>(-8.0, -2.1)<br>0.001	-4.6 (1.5)<br>(-7.5, -1.7)<br>.002	0.5 (1.5)<br>(-2.4, 3.3)<br>.759
Diastolic blood pressure, mm Hg	1.0 (2.5)	-0.8 (2.5)	-3.5 (2.4)	-4.5 (1.1)<br>(-6.7, -2.3)<br><.0001	-2.8 (1.1)<br>(-5.0, -0.5)<br>.015	1.7 (1.1)<br>(-0.5, 3.9)<br>.126
Total cholesterol, mmol/L	-0.09 (0.24)	0.17 (0.24)	-0.1 (0.24)	-0.01 (0.11)<br>(-0.23, 0.21)<br>.945	-0.26 (0.11)<br>(-0.48, -0.04)<br>.02	-0.26 (0.11)<br>(-0.47, 0.04)<br>.022

© 2024 Guo L et al. *JAMA Network Open.*

Triglycerides, mmol/L	-0.13 (0.48)	-0.12 (0.48)	-0.74 (0.46)	-0.6 (0.22)<br>(-1.04, -0.17)<br>.007	-0.62 (0.22)<br>(-1.05, -0.18)<br>.006	-0.01 (0.22)<br>(-0.44, 0.42)<br>.959
LDL cholesterol, mmol/L	-0.19 (0.20)	0.03 (0.20)	-0.11 (0.19)	0.08 (0.09)<br>(-0.10, 0.26)<br>.365	-0.14 (0.09)<br>(-0.31, 0.04)<br>.128	-0.22 (0.09)<br>(-0.39, 0.05)<br>.014
HDL cholesterol, mmol/L	0.13 (0.07)	0.2 (0.07)	0.24 (0.07)	0.06 (0.03)<br>(0.01, 0.11)<br>.02	0.06 (0.03)<br>(0.01, 0.11)<br>.024	0 (0.03)<br>(-0.05, 0.05)<br>.941
Uric acid, mmol/L	9.02 (19.35)	-34.54 (19.36)	-7.03 (18.75)	-16.05 (8.96)<br>(-33.66, 1.56)<br>.074	27.52 (8.94)<br>(9.94, 45.09)<br>.002	43.56 (8.80)<br>(26.25, 60.87)<br><.0001

Abbreviations: HbA_{1c}, glycated hemoglobin; HDL, high-density lipoprotein cholesterol; HOMA-IR, homeostasis model assessment of insulin resistance; LDL, low-density lipoprotein cholesterol.

Data are adjusted LSM (SE).

[a] The following covariates were used for adjustment in this analysis: sex, age, height, weight, family history of diabetes and hypertension, physical activity, smoking, alcohol consumption, and baseline.

© 2024 Guo L et al. *JAMA Network Open.*

eTable 5. Unadjusted[a] Analyses of Outcomes From Baseline to Week 16 in the Three Groups

	**Metformin (n=134)**	**Empagliflozin (n=136)**	**5:2 MR (n=135)**	**Treatment Comparison** *P* value		
				5:2 MR *vs* Metformin	5:2 MR *vs* Empagliflozin	Metformin *vs* Empagliflozin
**Primary endpoint**						
HbA_{1c}, %	-1.49 (0.97)	-1.32 (0.88)	-1.81 (0.92)	.017	<.001	.343
**Secondary endpoints**						
Bodyweight, kg	-4.1 (8.3)	-5.0 (8.2)	-9.6 (10.1)	<.0001	<.0001	1
Body mass index, kg/m^2	-1.46 (2.84)	-1.78 (2.82)	-3.39 (3.49)	<.0001	<.0001	1
Fasting plasma glucose, mmol/L	-1.25 (2.26)	-1.31 (2.29)	-1.76 (2.11)	.181	.271	1
Fasting insulin, µU/mL	-2.15 (30.75)	-2.17 (35.2)	-13.67 (42.89)	.031	.03	1
Fasting C-peptide, ng/mL	-0.4 (1.64)	-0.59 (3.1)	-0.67 (1.32)	.935	1	1
HOMA-IR	-2.25 (16.82)	-1.71 (19.76)	-7.1 (23.17)	.144	.083	1
Waist circumference, cm	-2.3 (7.4)	-2.0 (7.9)	-5.0 (8.8)	<.0001	<.0001	1
Hip circumference, cm	-3.5 (2.1)	-3.4 (2.1)	-5.5 (2.1)	.017	.008	1

© 2024 Guo L et al. *JAMA Network Open.*

Waist-to-hip ratio	-0.03 (0.16)	-0.03 (0.18)	-0.06 (0.17)	.474	.322	1
Systolic blood pressure, mm Hg	-3.1 (14.7)	-4.2 (14.8)	-8.7 (13.9)	.005	.032	1
Diastolic blood pressure, mm Hg	-0.7 (10.7)	-2.6 (11.3)	-5.6 (10.9)	.001	.075	.445
Total cholesterol, mmol/L	-0.22 (0.96)	0.05 (1.33)	-0.15 (0.99)	1	.416	.139
Triglycerides, mmol/L	-0.23 (2.28)	-0.14 (3.14)	-0.56 (1.9)	.867	.514	1
LDL cholesterol, mmol/L	-0.23 (0.76)	0.01 (0.92)	-0.12 (0.8)	.868	.511	.046
HDL cholesterol, mmol/L	0.09 (0.21)	0.1 (0.21)	0.14 (0.22)	.12	.322	1
Uric acid, mmol/L	17.35 (97.21)	-33.96 (98.56)	-1.3 (97.32)	.355	.019	<.001

Abbreviations: HbA_{1c}, glycated hemoglobin; HDL, high-density lipoprotein cholesterol; HOMA-IR, homeostasis model assessment of insulin resistance; LDL, low-density lipoprotein cholesterol.

Data are mean (SD).

[a] All patients in the full analysis set are included in the treatment comparisons, without adjustment for the following baseline characteristics (sex, age, height, weight, family history of diabetes and hypertension, physical activity, smoking, alcohol consumption, and baseline HbA_{1c}).

© 2024 Guo L et al. *JAMA Network Open.*

eTable 6. Primary and Secondary Outcomes from Baseline to Week 16 in Three Groups (Completers Analysis)

	n	n_{miss}	16 weeks	Treatment Comparison LSM difference (SE); 95% CI; *P* value		
				5:2 MR *vs* Metformin	5:2 MR *vs* Empagliflozin	Metformin *vs* Empagliflozin
**Primary endpoint**						
HbA_{1c}, %						
Metformin	115	19	-1.65 (0.19)	-0.35 (0.1)	-0.56 (0.10)	-0.21 (0.10)
Empagliflozin	107	29	-1.44 (0.19)	(-0.54, -0.16)	(-0.75, -0.36)	(-0.40, 0.02)
5:2 MR	110	25	-2.00 (0.19)	<.0001	<.0001	.03
**Secondary endpoints**						
Bodyweight, kg						
Metformin	114	20	-4.9 (1.2)	-6.1 (0.6)	-5.5 (0.6)	0.6 (0.6)
Empagliflozin	107	29	-5.5 (1.2)	(-7.2, -5.0)	(-6.65, -4.4)	(-0.5, 1.7)
5:2 MR	110	25	-11.0 (1.1)	<.0001	<.0001	.292
Body mass index, kg/m^2						
Metformin	114	20	-1.63 (0.42)	-2.18 (0.20)	-1.94 (0.21)	0.25 (0.20)
Empagliflozin	107	29	-1.87 (0.42)	(-2.58, -1.79)	(-2.34, -1.53)	(-0.14, 0.64)
5:2 MR	110	25	-3.81 (0.40)	<.0001	<.0001	.214
Fasting plasma glucose, mmol/L						
Metformin	112	23	-1.27 (0.33)	-0.78 (0.17)	-0.58 (0.17)	0.20 (0.17)
Empagliflozin	104	32	-1.47 (0.33)	(-1.11, -0.45)	(-0.91, -0.25)	(-0.12, 0.52)

© 2024 Guo L et al. *JAMA Network Open.*

5:2 MR	109	26	-2.04 (0.32)	<.0001	.001	.225
Waist circumference, cm						
Metformin	113	21	-4.3 (1.4)	-7.1 (0.7)	-6.6 (0.7)	0.4 (0.7)
Empagliflozin	107	28	-4.6 (1.4)	(-8.4, -5.8)	(-8.0, -5.3)	(-0.9, 1.7)
5:2 MR	110	25	-11.2 (1.3)	<.0001	<.0001	.511
Hip circumference, cm						
Metformin	113	21	-2.5 (1.1)	-4.3 (0.5)	-3.8 (0.5)	-0.4 (0.5)
Empagliflozin	107	28	-2.9 (1.1)	(-5.3, -3.2)	(-4.9, -2.8)	(-0.6, 1.4)
5:2 MR	110	25	-6.7 (1.0)	<.0001	<.0001	.40
Waist-to-hip ratio						
Metformin	113	21	-0.02 (0.02)	-0.03 (0.01)	-0.03 (0.01)	0.01 (0.01)
Empagliflozin	107	28	-0.02 (0.02)	(-0.05, -0.02)	(-0.05, -0.01)	(-0.01, 0.02)
5:2 MR	110	25	-0.05 (0.02)	.001	.005	.547
Systolic blood pressure, mm Hg						
Metformin	114	20	-0.7 (3.0)	-6.6 (1.5)	-4.9 (1.6)	1.7 (1.5)
Empagliflozin	106	30	-2.4 (3.1)	(-9.6, -3.6)	(-8.0, 1.9)	(-1.3, 4.6)
5:2 MR	110	25	-7.3 (2.9)	<.0001	.002	.277
Diastolic blood pressure, mm Hg						
Metformin	114	20	1.3 (2.4)	-5.3 (1.2)	-3.2 (1.2)	2.1 (1.2)
Empagliflozin	106	30	-0.8 (2.4)	(-7.7, -2.9)	(-5.6, -0.8)	(-0.3, 4.4)
5:2 MR	110	25	-4.0 (2.3)	<.0001	.01	.085

© 2024 Guo L et al. *JAMA Network Open.*

Total cholesterol, mmol/L						
Metformin	112	22	-0.07 (0.23)	-0.09 (0.12)	-0.35 (0.12)	-0.26 (0.12)
Empagliflozin	105	31	0.19 (0.23)	(-0.33, 0.14)	(-0.59, -0.12)	(-0.49, -0.03)
5:2 MR	109	26	-0.16 (0.22)	.424	.004	.028
Triglycerides, mmol/L						
Metformin	112	22	-0.15 (0.43)	-0.92 (0.22)	-0.92 (0.22)	-0.01 (0.22)
Empagliflozin	105	31	-0.15 (0.43)	(-1.35, -0.49)	(-1.36, -0.49)	(-0.43, 0.42)
5:2 MR	109	26	-1.07 (0.42)	<.0001	<.0001	.982
LDL cholesterol, mmol/L						
Metformin	112	22	-0.15 (0.19)	0.04 (0.10)	-0.12 (0.10)	-0.16 (0.10)
Empagliflozin	105	31	0.02 (0.20)	(-0.15, 0.24)	(-0.32, 0.08)	(-0.35, 0.03)
5:2 MR	109	26	-0.10 (0.19)	.673	.23	.097
HDL cholesterol, mmol/L						
Metformin	112	22	0.12 (0.05)	0.07 (0.02)	0.06 (0.03)	-0.01 (0.02)
Empagliflozin	105	31	0.13 (0.05)	(0.02, 0.12)	(0.01, 0.11)	(-0.05, 0.04)
5:2 MR	109	26	0.19 (0.05)	.005	.013	.802
Uric acid, mmol/L						
Metformin	112	22	12.64 (20.30)	-24.64 (10.26)	35.45 (10.49)	60.08 (10.22)
Empagliflozin	105	31	-47.45 (20.45)	(-44.83, -4.45)	(14.81, 56.08)	(39.98, 80.19)
5:2 MR	109	26	-12.00 (19.62)	.017	.001	<.0001

Abbreviations: HbA_{1c}, glycated hemoglobin; HDL, high-density lipoprotein cholesterol; LDL, low-density lipoprotein cholesterol.

Data are LSM (SE).

© 2024 Guo L et al. *JAMA Network Open.*

eTable 7. Primary and Secondary Outcomes from Baseline to Week 24 in Three Groups (Completers Analysis)

	**n**	**n_{miss}**	**24 weeks**	**Treatment Comparison** LSM difference (SE); 95% CI; P value		
				5:2 MR *vs* Metformin	5:2 MR *vs* Empagliflozin	Metformin *vs* Empagliflozin
**Primary endpoint**						
HbA_{1c}, %						
Metformin	96	38	-1.32 (0.20)	-0.56 (0.11)	-0.66 (0.11)	-0.10 (0.11)
Empagliflozin	87	49	-1.22 (0.20)	(-0.78, -0.35)	(-0.88, -0.45)	(-0.31, 0.11)
5:2 MR	94	41	-1.88 (0.19)	<.0001	<.0001	.36
**Secondary endpoints**						
Bodyweight, kg						
Metformin	97	37	-4.0 (1.4)	-5.5 (0.7)	-4.8 (0.8)	0.6 (0.7)
Empagliflozin	88	48	-4.6 (1.4)	(-6.9, -4.0)	(-6.3, -3.4)	(-0.8, 2.0)
5:2 MR	92	43	-9.4 (1.3)	<.0001	<.0001	.407
Body mass index, kg/m^2						
Metformin	97	37	-1.99 (0.99)	-1.16 (0.51)	-1.82 (0.53)	-0.66 (0.51)
Empagliflozin	88	48	-1.33 (1.00)	(-2.17, -0.15)	(-2.86, -0.77)	(-1.67, 0.34)
5:2 MR	92	43	-3.15 (0.95)	.025	.001	.193
Fasting plasma glucose, mmol/L						
Metformin	96	38	-0.80 (0.35)	-1.07 (0.19)	-0.70 (0.19)	0.37 (0.19)

© 2024 Guo L et al. *JAMA Network Open.*

Empagliflozin	86	50	-1.17 (0.35)	(-1.44, -0.70)	(-1.08, 0.32)	(0, 0.74)
5:2 MR	94	41	-1.87 (0.33)	<.0001	<.0001	.051
Waist circumference, cm						
Metformin	97	37	-4.3 (1.6)	-6.4 (0.8)	-5.9 (0.8)	0.6 (0.8)
Empagliflozin	88	48	-4.9 (1.6)	(-8.0, -4.8)	(-7.4, -4.2)	(-1.0, 2.2)
5:2 MR	91	44	-10.7 (1.5)	<.0001	<.0001	.435
Hip circumference, cm						
Metformin	97	37	-2.4 (1.1)	-3.8 (0.6)	-4.2 (0.6)	-0.5 (0.6)
Empagliflozin	88	48	-2.0 (1.1)	(-4.9, -2.6)	(-5.4, -3.0)	(-1.6, 0.7)
5:2 MR	91	44	-6.2 (1.1)	<.0001	<.0001	.445
Waist-to-hip ratio						
Metformin	97	37	-0.03 (0.03)	-0.02 (0.02)	-0.03 (0.02)	-0.01 (0.02)
Empagliflozin	88	48	-0.02 (0.03)	(-0.05, 0.01)	(-0.07, 0.00)	(-0.04, 0.02)
5:2 MR	91	44	-0.05 (0.03)	.181	.056	.515
Systolic blood pressure, mm Hg						
Metformin	97	37	-11.5 (3.4)	-6.0 (1.9)	-3.1 (1.9)	2.9 (1.9)
Empagliflozin	86	50	-14.4 (3.4)	(-9.6, -2.3)	(-6.8, 0.7)	(-0. 8, 6.5)
5:2 MR	92	43	-17.4 (3.3)	.001	.107	.123
Diastolic blood pressure, mm Hg						
Metformin	97	37	-2.8 (2.3)	-3.7 (1.2)	-3.1 (1.3)	0.63 (1.22)
Empagliflozin	86	50	-3.4 (2.3)	(-6.1, -1.3)	(-5.6, -0.6)	(-1.78, 3.03)
5:2 MR	92	43	-6.5 (2.2)	.003	.015	.609

© 2024 Guo L et al. *JAMA Network Open.*

Total cholesterol, mmol/L						
Metformin	96	38	0.03 (0.23)	-0.19 (0.13)	-0.36 (0.13)	-0.18 (0.13)
Empagliflozin	87	49	0.20 (0.24)	(-0.44, 0.07)	(-0.62, -0.11)	(-0.43, 0.08)
5:2 MR	94	41	-0.16 (0.22)	.146	.006	.169
Triglycerides, mmol/L						
Metformin	96	38	-0.06 (0.52)	-0.80 (0.28)	-0.68 (0.29)	0.12 (0.29)
Empagliflozin	87	49	-0.17 (0.53)	(-1.36, -0.26)	(-1.26, -0.11)	(-0.45, 0.68)
5:2 MR	94	41	-0.86 (0.50)	.005	.019	.685
LDL cholesterol, mmol/L						
Metformin	95	39	0.02 (0.21)	-0.15 (0.11)	-0.18 (0.12)	-0.03 (0.11)
Empagliflozin	86	50	0.06 (0.21)	(-0.37, 0.07)	(-0.40, 0.05)	(-0.25, 0.19)
5:2 MR	94	41	-0.12 (0.20)	.192	.122	.78
HDL cholesterol, mmol/L						
Metformin	95	39	0.13 (0.05)	0.09 (0.03)	0.05 (0.03)	-0.04 (0.03)
Empagliflozin	86	50	0.17 (0.05)	(0.04, 0.15)	(0, 0.11)	(-0.09, 0.01)
5:2 MR	94	41	0.22 (0.05)	.001	.056	.13
Uric acid, mmol/L						
Metformin	96	38	14.15 (19.18)	-13.04 (10.39)	-4.44 (10.66)	8.60 (10.47)
Empagliflozin	87	39	5.55 (19.34)	(-33.50, 7.42)	(-25.43, 16.55)	(-12.02, 29.22)
5:2 MR	94	41	1.11 (18.41)	.211	.678	.412

Abbreviations: HbA_{1c}, glycated hemoglobin; HDL, high-density lipoprotein cholesterol; LDL, low-density lipoprotein cholesterol.

Data are LSM (SE).

© 2024 Guo L et al. *JAMA Network Open.*

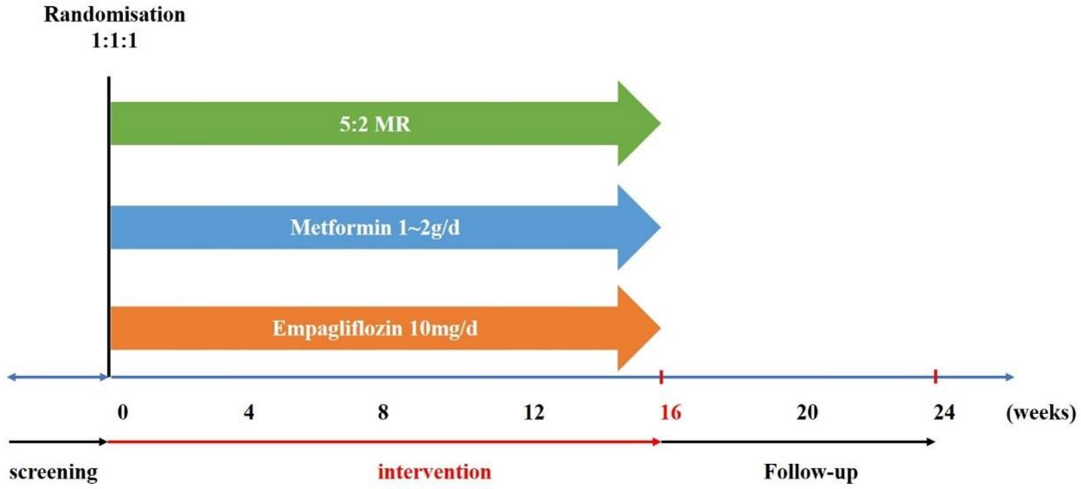

eFigure 1. Study design. Participants were randomly assigned to the metformin, empagliflozin, or 5:2 MR groups for a 16-week treatment. After the 16-week treatment, the participants in all three groups underwent an additional 8-week follow-up without any intervention, i.e., the final visit was conducted at week 24.

© 2024 Guo L et al. *JAMA Network Open.*

5:2 intermittent fasting meal replacement diet Group (5+2 MR)		
	**On the 2 light fasting days**	**On the 5 non-fasting days:**
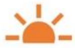	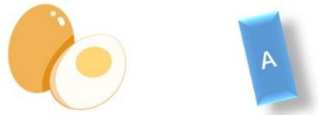	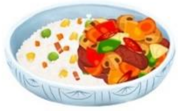
	1 egg + one serving Kang Zhijun™ A	Regular meals
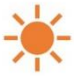	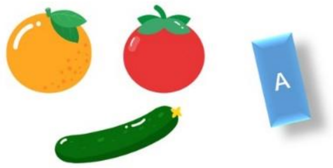	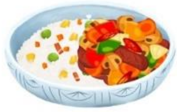
	100g of low-carbohydrate fruits or vegetables (such as cucumber, tomatoes, oranges, etc.) + one serving Kang Zhijun™ A	Regular meals
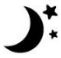	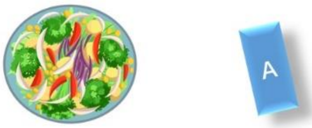	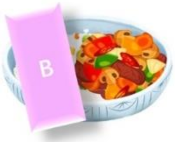
	200~400g of green leafy vegetables (such as spinach, cabbage, etc.) + one serving Kang Zhijun™ A	one serving Kang Zhijun™ B instead of carbohydrate-rich foods+ Other foods

eFigure 2. The 5:2 MR approach. Participant are required to choose *2* non-consecutive days within a week (such as Monday and Thursday, or Tuesday and Friday) to replace all three meals with Kang zhijun™ A. The dietary plan for these two days is as follows as example: one egg for breakfast + Kang zhijun™ A, 100g of low-sugar fruit (such as cucumber, tomato and orange) for lunch + Kang zhijun™ A, and 200-400g of green leafy vegetables (such as spinach and cabbage) for dinner + Kang zhijun™ A. The specified total daily energy intake is 500 kcal for females and 600 kcal for males per day. For the remaining *5* days of the week, breakfast and lunch should be regular meals (less intake of oil and sugar is recommended), and dinner will be replaced with Kang zhijun™ B.

© 2024 Guo L et al. *JAMA Network Open.*

A

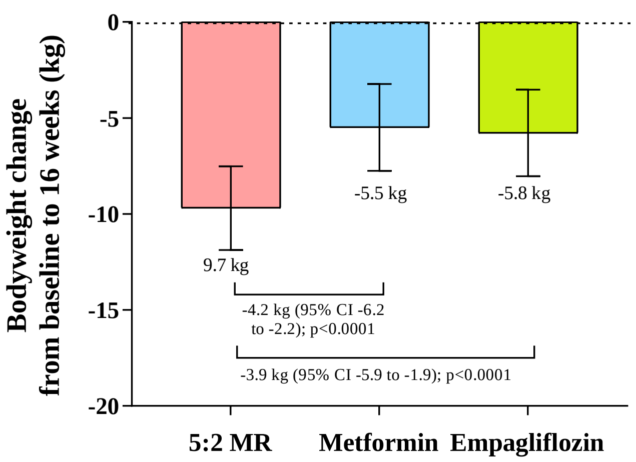

B

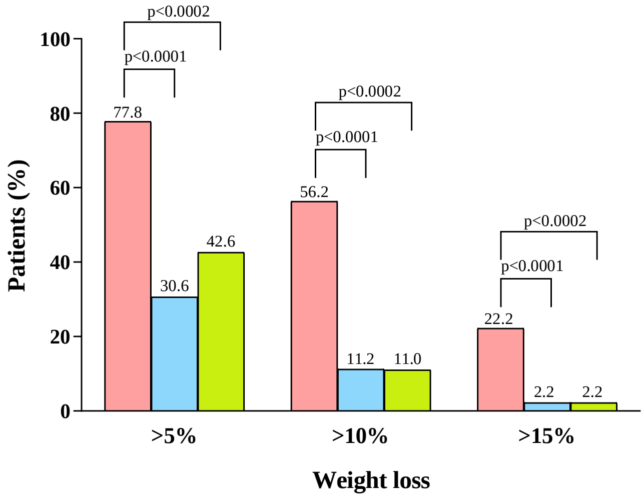

eFigure 3. Changes in Body Weight. (A) Changes in body weight from baseline to 16 weeks. (B) Proportions of patients with 5%, 10%, and 15% weight loss in all three groups.

© 2024 Guo L et al. *JAMA Network Open.*

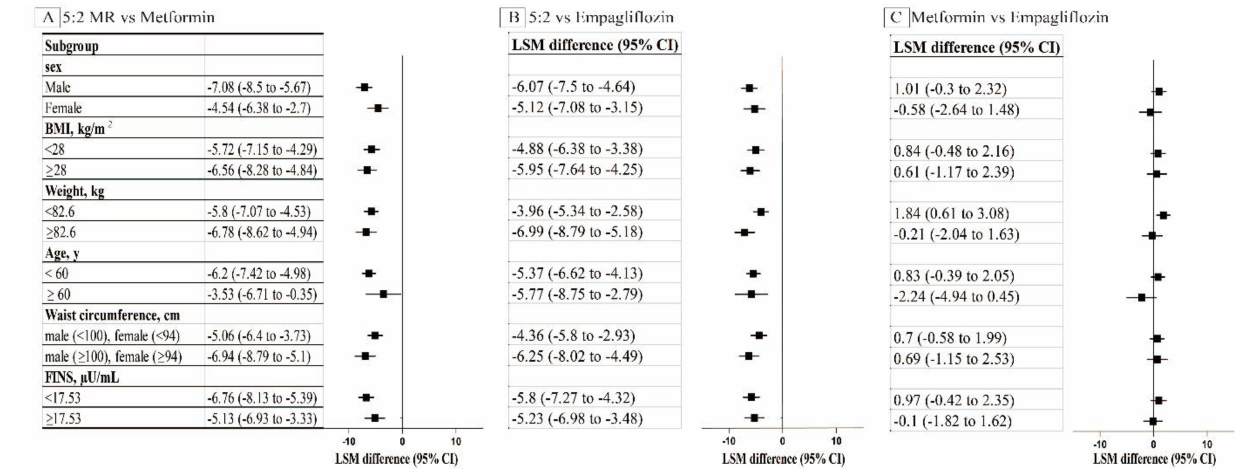

eFigure 4. Post-Hoc Subgroup Analysis of Weight Loss at Week 16 by Intention-to-Treat Analysis. Patients were randomized to receive 5:2MR (n=135), metformin (n=134) and empagliflozin (n=136). Subgroups are categorized by sex (male, female), BMI (≥24, <28 kg/m^2, ≥28 kg/m^2), weight (<82.6 kg, ≥ 82.6 kg), age (<60 years, ≥60 years), waist circumference (male: <100 cm, ≥100 cm; female: <94 cm, ≥94 cm), and fasting insulin (≤17.53 µU/ml, >17.53 µU/ml).

© 2024 Guo L et al. *JAMA Network Open.*

eReference.

1. Dai X, Deng WP, Dong YX, et al. China Diabetes Care and Education Guidelines. https://diab.cma.org.cn/UploadFile/Ueditor/file/20160811/6360650900034000003924937.pdf
