# Supplementary material for: A 5:2 Intermittent Fasting Meal Replacement Diet and Glycemic Control for Adults With Diabetes: The EARLY Randomized Clinical Trial
Source: JAMA Netw Open. 2024 Jun 21;7(6):e2416786. doi: 10.1001/jamanetworkopen.2024.16786 (PMC11193124; doi:10.1001/jamanetworkopen.2024.16786)
Supplement: Supplement 3. — Data Sharing Statement [file jamanetwopen-e2416786-s003.pdf]

Data Sharing Statement

Guo. A 5:2 Intermittent Fasting Meal Replacement Diet and Glycemic Control for Adults With Diabetes. *JAMA Netw Open*. Published June 21, 2024.
doi:10.1001/jamanetworkopen.2024.16786

Data
Data available: Yes
Data types: Deidentified participant data
How to access data: Requests for data can be sent to glx1218@163.com
When available: With publication

Supporting Documents
Document types: None

Additional Information
Who can access the data: Researchers whose proposed use of the data has been approved.
Types of analyses: For any research purpose
Mechanisms of data availability: With a signed Data Use Agreement.
